# Oxidative stress induces cell death partially by decreasing both mRNA and protein levels of nicotinamide phosphoribosyltransferase in differentiated PC12 cells

**DOI:** 10.7717/peerj.11401

**Published:** 2021-05-14

**Authors:** Cuiyan Zhou, Weihai Ying

**Affiliations:** Med-X Research Institute and School of Biomedical Engineering, Shanghai Jiao Tong University, Shanghai, China

**Keywords:** Nicotinamide phosphoribosyltransferase, NAD^+^, Oxidative stress, Cell death, Aging

## Abstract

**Background.:**

Multiple studies have indicated crucial roles of NAD^+^ deficiency in several neurological diseases and aging. It is critical to discover the mechanisms underlying the NAD^+^ deficiency. A decreased level of Nicotinamide phosphoribosyltransferase (Nampt)—an important enzyme in the salvage pathway of NAD^+^ synthesis—has been found under certain pathological conditions, while the mechanisms underlying the Nampt decrease are unclear. The purpose of this study is to test the hypothesis that oxidative stress can produce decreased Nampt, and to investigate the biological effects of Nampt on NAD^+^ synthesis and cell survival under both basal and oxidative stress conditions.

**Methods.:**

We used differentiated PC12 cells as a cellular model to investigate the effects of oxidative stress on the levels of Nampt. Multiple assays, including flow cytometry-based cell death assays and NAD^+^ assays were conducted.

**Results.:**

First, oxidative stress can decrease the levels of Nampt mRNA and Nampt protein; second, Nampt plays significant roles in NAD^+^ synthesis under both basal conditions and oxidative stress conditions; third, Nampt plays critical roles in cell survival under both basal conditions and oxidative stress conditions; and fourth, oxidative stress produced decreased NAD^+^ levels and cell survival partially by decreasing Nampt. Collectively, our study has indicated that oxidative stress is a pathological factor leading to decreased Nampt, which plays important roles in oxidative stress-produced decreases in NAD^+^ levels and cell survival. Our findings have indicated major roles of Nampt in maintaining NAD^+^ levels and cell survival under both basal and oxidative stress conditions.

## Introduction

Cumulative evidence has domonstrated that NAD^+^ plays significant roles in multiple biological processes (Ying, 2008). Multiple studies have also suggested that NAD^+^ deficiency is a pivotal pathological factor in numerous major diseases and pathological conditions (Ying, 2008; Ying, 2006; Zhang & Ying, 2019), including ischemic brain injury (Ying et al., 2007; Zheng et al., 2012), myocardial ischemia (Zhang et al., 2016), head trauma (Won et al., 2012), epilepsy (Liu et al., 2017), radiation-induced tissue injury (Sheng et al., 2012) and chemotherapy agent-induced tissue injury (Wang et al., 2014). NAD^+^ deficiency also appears to play an important role in the aging process (Frederick et al., 2016; Mills et al., 2016; Son et al., 2016), which has been the biological basis for the wide applications of NAD^+^ and NAD^+^ precursors for delaying aging. It has critical theoretical and medical importance to expose the impact of NAD^+^ metabolism on pathological alterations. However, there have been no sufficient studies on this critical topic.

The salvage pathway and the de novo pathway are two major pathways for NAD^+^ synthesis (Magni et al., 2004). The nuclear enzyme Nicotinamide mononucleotide adenylyltransferase-1 (NMNAT-1) is a crucial enzyme in the two pathway of NAD^+^ synthesis (Magni et al., 2004). Mammals use nicotinamide as the precursor for NAD^+^ synthesis in the salvage pathway (Revollo, Grimm & Imai, 2007): nicotinamide phosphoribosyltransferase (Nampt) converts nicotinamide to nicotinamide mononucleotide that can be converted to NAD^+^ by NMNATs (Revollo, Grimm & Imai, 2007).

NAD^+^ deficiency has been observed in a variety of disease models, which may result from poly (ADP-ribose) polymerase-1 activation or reduced Nampt activity. It has been indicated that decreased Nampt activity is an important mechanism accounting for declined NAD^+^ synthesis: Decreased Nampt activity has been found in the metabolic organs that are exposed to high-fat diet (Yoshino et al., 2011), Drosophila pink1 mutants (Lehmann, Loh & Martins, 2017), and aging (Jokinen et al., 2017; Yoshino et al., 2011). It is necessary to investigate the potential mechanisms for the decreased Nampt levels.

Our study used PC12 cells to investigate the effects of oxidative stress on both the Nampt mRNA and Nampt protein levels. We also investigated the roles of Nampt in the cell death and NAD^+^ metabolism under both basal and oxidative stress conditions. Our experimental results have shown that oxidative stress can produce decreased levels of Nampt, which contributes to oxidative stress-produced decreases in NAD^+^ levels and cell survival. Our current study has also indicated that Nampt plays important roles in maintaining intracellular NAD^+^ levels and cell survival under both basal and oxidative stress conditions.

## Materials and Methods

### Materials

Sigma–Aldrich (St. Louis, MO, USA) was the supplier of H_2_O_2_ (323,381) and Nicotinamide (72,340). FK866 (HY-50876) and P7C3 (HY-15976) were purchased from MedChemExpress (Monmouth Junction, NJ, USA). Nampt siRNAs and control siRNAs were purchased from GenePharma (Shanghai, China). Sangon Biotech (Shanghai, China) was the supplier of Nampt primers.

### Cell cultures

We purchased differentiated PC12 cells from the Cell Resource Center of Shanghai Institute of Biological Sciences, Chinese Academy of Sciences (Shanghai, China). PC12 cells were plated into 12- well or 24-well cell culture plates in DMEM (Hyclone, MA, USA) containing 10% FBS, 1% penicillin and 1% streptomycin. The cells were cultured in an incubator with 5% CO_2_ and the culture temperature was 37 °C.

### NAD^+^ assay

As previously described (Alano, Ying & Swanson, 2004), recycling assay was conducted to determine the level of intracellular NAD^+^: cells were lysed by perchloric acid. Samples were centrifugated at 12,000×*g* for 5 min, and the supernatant was transferred to new test tubes. A total of 3 N potassium hydroxide and 1 M potassium phosphate buffer were used to neutralize the samples to pH 7.2. After centrifugation, the samples were mixed with reaction buffer that contained 1.7 mg 3-[4,5-dimethylthiazol-2-yl]-2,5-diphenyl-tetrazolium bromide (MTT), 488.4 mg nicotinamide, 10.6 mg phenazine methosulfate,1.3 mg alcohol dehydrogenase, and 2.4 mL ethanol in 37.6 mL Gly-Gly buffer (65 mM, pH 7.4). A total of 10 min later, the A_560nm_ of the samples was assessed. The final concentrations of NAD^+^ were normalized by protein levels.

### Annexin V/7-AAD assay

Flow cytometry assay was conducted to determine the levels of necrosis and apoptosis of PC12 cells, and the ApoScreen Annexin V kit (SouthernBiotech, Birmingham, AL, USA) was used. Briefly, differentiated PC12 cells were harvested by 0.25% trypsin solution. After washes with PBS, the cells were incubated with 100 μL cold 1X binding buffer and 5 μL Annexin V, which were put in ice for 15 min. Finally, 200 μL 1X binding buffer and 5 μL 7-AAD were mixed into the samples. The levels of necrosis and apoptosis of PC12 cells were measured by a flow cytometer (FACSAria II, BD Biosciences).

### Real-time PCR assay

The total RNA of PC12 cells was harvested by TaKaRa RNA Extraction Kit (Takara Bio, Dalian, China) according to the manufacturer’s protocol. cDNA was reverse-transcribed from 500 ng total RNA using a Prime-Script RT reagent kit produced by Takara Bio (Dalian, China). The reverse transcription was conducted as follows: 37 °C for 15 min, followed by 85 °C for 15 s. SYBR Premix Ex Taq produced by Takara Bio (Dalian, China) was used for conducting Quantitative RT-PCR, in which the following primers were used: Nampt (sense 5′- TATTCTGTTCCAGCGGCAGA -3′ and anti-sense 5′- GACCACAGACACAGGCACTGA -3′) ; GAPDH (sense 5′- CCTGCACCACCAACTGCTTA -3′ and anti-sense 5′- GGCCATCCACAGTCTTCTGA -3′). The following procedure was conducted for the assays: after denaturation at 95 °C for 10 s, 40 cycles of 95 °C for 5 s and 60° C for 30 s were conducted. The comparative threshold cycle method was used for the data analyses. The results were normalized by GAPDH mRNA level.

### Western blot assay

After treatment, PC12 cells were washed twice with PBS. The cells were lysed in RIPA buffer produced by Millipore (Temecula, CA, USA) which contained 1 mM phenylmethanesulfonyl fluoride and 1% protease inhibitor cocktail produced by CWBio (Beijing, China). After centrifugation at 12,000 rpm for 10 min at 4 °C, the protein concentrations of the samples were determined by BCA Protein Assay Kit (Pierce Biotechnology, Rockford, IL, USA). Thirty μg of the sample was electrophoresed through a 10% sodium dodecyl sulfate-polyacrylamide gel and then transferred to 0.45-μm nitrocellulose membranes. The blots were incubated with a polyclonal Nampt antibody from Abcam (Cambridge, UK), which was diluted in TBST containing 1% bovine serum albumin at 1:2,000 ratio, at 4 °C overnight. Subsequently, a horse radish peroxidase-conjugated secondary antibody produced by Epitomics (Zhejiang Province, China) diluted by TBST containing 1% bovine serum albumin at 1:3,000 ratio was used for incubation of the blots for 1 h at room temperature. The normalization of the sample was conducted by using a Tubulin antibody produced by proteintech (Wuhan, China). Quantifications of the bands were performed by Gel-Pro Analyzer (Media Cybernetics, Silver Spring, MD, USA).

### RNA interference

PC12 cells at the density of approximately 40% were transfected with he scrambled control siRNA sequences and Nampt siRNA with the following sequences: sense 5′-AGUAAGGAAGGUGAAAUACTT-3′ and antisense 5′- GUAUUUCACCUUCCUUACUTT-3′. The transfection reagent used was lipofectamine 2000 (Invitrogen, Carlsbad, CA, USA). Each well contained 2.5 µl lipofectamine 2000, 0.06 nmol siRNA sequences,100 µl Opti-MEM, and 500 µl Dulbecco’s modified Eagle medium. After 6 h, the media was replaced by Dulbecco’s modified Eagle medium containing 10% FBS.

### Statistical analyses

Data were shown as mean ± SEM, which were analyzed by one-way ANOVA or two-way ANOVA. Student—Newman—Keuls post hoc tests were used.

## Results

### Nampt inhibitor FK866 significantly decreased the intracellular NAD^+^ level and cell survival of PC12 cells under basal conditions

**Figure 1:**
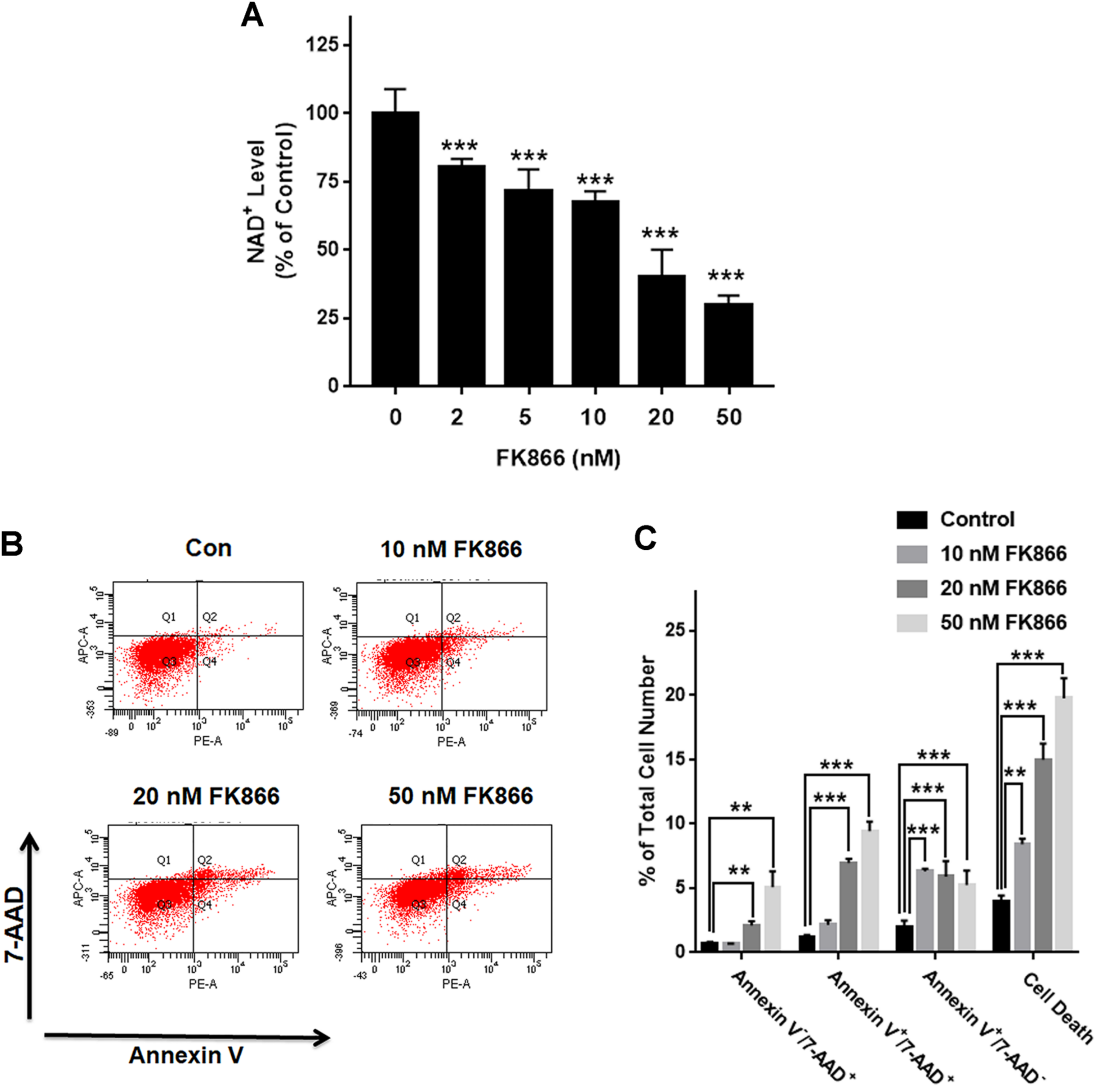
Nampt plays a siginificant role in the NAD^+^ synthesis and basal survival of differentiated PC12 cells. (A) ****FK866 at the concentrations of 2, 5, 10, 20, and 50 nM produced dose-dependent decreases in the intracellular NAD^+^ levels. NAD^+^ assays were conducted 6 h after the cells were treated with 2, 5, 10, 20, or 50 nM FK866. The data were pooled from three independent experiments. *N* = 3. There were three, three and three samples in these three independent experiments. ***P < 0.001. (B, C) Ten nM FK866 led to a significant increase in early-stage apoptosis, while both 20 nM and 50 nM FK866 led to significant increases in early-stage apoptosis (Annexin V^+^/7-ADD^−^), late-stage apoptosis (Annexin V^+^/7-ADD^+^) and necrosis (Annexin V^−^/7-ADD^+^). PC12 cells were treated with 10, 20, or 50 nM FK866 for 24 h. The data were pooled from three independent experiments. *N* = 3. There were 3, 3 and 3 samples in these three independent experiments. **P < 0.01; ***P < 0.001.

We investigated the roles of Nampt in maintaining the NAD^+^ level of PC12 cells under basal conditions. Nampt inhibitor FK866 was used to attenuate the activity of Nampt. Our experimental results showed that FK866 at the concentrations of 2, 5, 10, 20, and 50 nM produced dose-dependent decreases in the NAD^+^ levels (Fig. 1A), indicating a critical role of Nampt in the NAD^+^ synthesis under basal conditions. We also conducted FACS-based Annexin V/7-ADD assay to evaluate the role of Nampt in the cell survival under basal conditions, showing that FK866 produced dose-dependent increases in cell death, which equals to the sum of early-stage apoptosis, late-stage apoptosis and necrosis (Figs. 1B and 1C).

### H_2_O_2_ produced significant decreases in both Nampt mRNA levels and Nampt protein levels of PC12 cells

**Figure 2:**
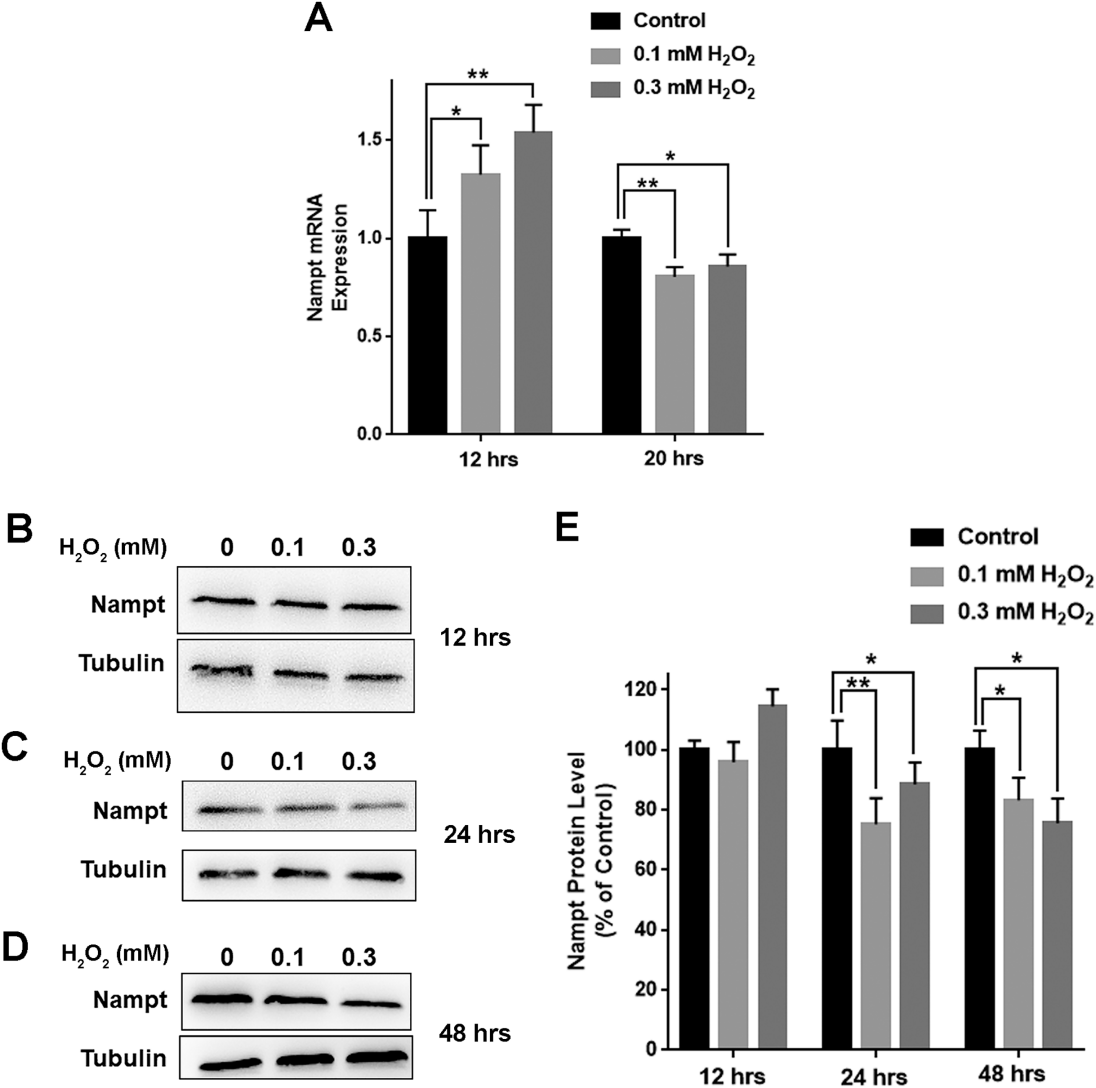
H_2_O_2_ produced significant decreases in both Nampt mRNA levels and Nampt protein levels of PC12 cells. ****(A) ****Treatment of PC12 cells with 0.1 mM or 0.3 mM ****H_2_O_2_ led to significant increases in the Nampt mRNA level of the cells 12 h after the H_2_O_2_ treatment. In contrast, H_2_O_2_ led to significant decreases in the Nampt mRNA level of the cells 20 h after the H_2_O_2_ treatment. PC12 cells were treated with 0.1 or 0.3 mM H_2_O_2_ for 12 or 20 h. The data were pooled from three independent experiments. *N* = 3. There were three, three and three samples in these three independent experiments. *P < 0.05; **P < 0.01. (B) Western blot assays showed the e ffects of H_2_O_2_ on the protein level of Nampt 12 h after the H_2_O_2_ treatment. (C) Western blot assays showed the effects of H_2_O_2_ on the protein level of Nampt 24 h after the H_2_O_2_ treatment. **(**D) Western blot assays showed the effects of H_2_O_2_ on the protein level of Nampt 48 h after the H_2_O_2_ treatment. (E) ****Quantifications of the Western blot assays showed that H_2_O_2_ led to significant decreases in the Nampt protein level of the cells 24 and 48 h, but not 12 h, after the H_2_O_2_ treatment. PC12 cells were treated with 0.1 or 0.3 mM H_2_O_2_ for 12, 24, or 48 h. The data were pooled from three independent experiments. *N* = 3. There were 3, 3 and 6 samples in these three independent experiments. *P < 0.05; **P < 0.01.

We further determined the impact of oxidative stress on the levels of Nampt mRNA and Nampt protein of PC12 cells: treatment of the cells with 0.1 or 0.3 mM H_2_O_2_ led to significant increases in the Nampt mRNA level of the cells 12 h after the treatment (Fig. 2A). In contrast, H_2_O_2_ led to decreases in the Nampt mRNA level 20 h after the treatment (Fig. 2A). H_2_O_2_ did not significantly affect the protein level of Nampt 12 h after the H_2_O_2_ treatment (Figs. 2B and 2E), while H_2_O_2_ led to significant decreases in the Nampt protein level both 24 h (Figs. 2C and 2E) and 48 h (Figs. 2D and 2E) after the H_2_O_2_ treatment.

### Decreased Nampt led to further decreases in the H_2_O_2_-produced reductions of the NAD^+^ levels and survival of PC12 cells

**Figure 3:**
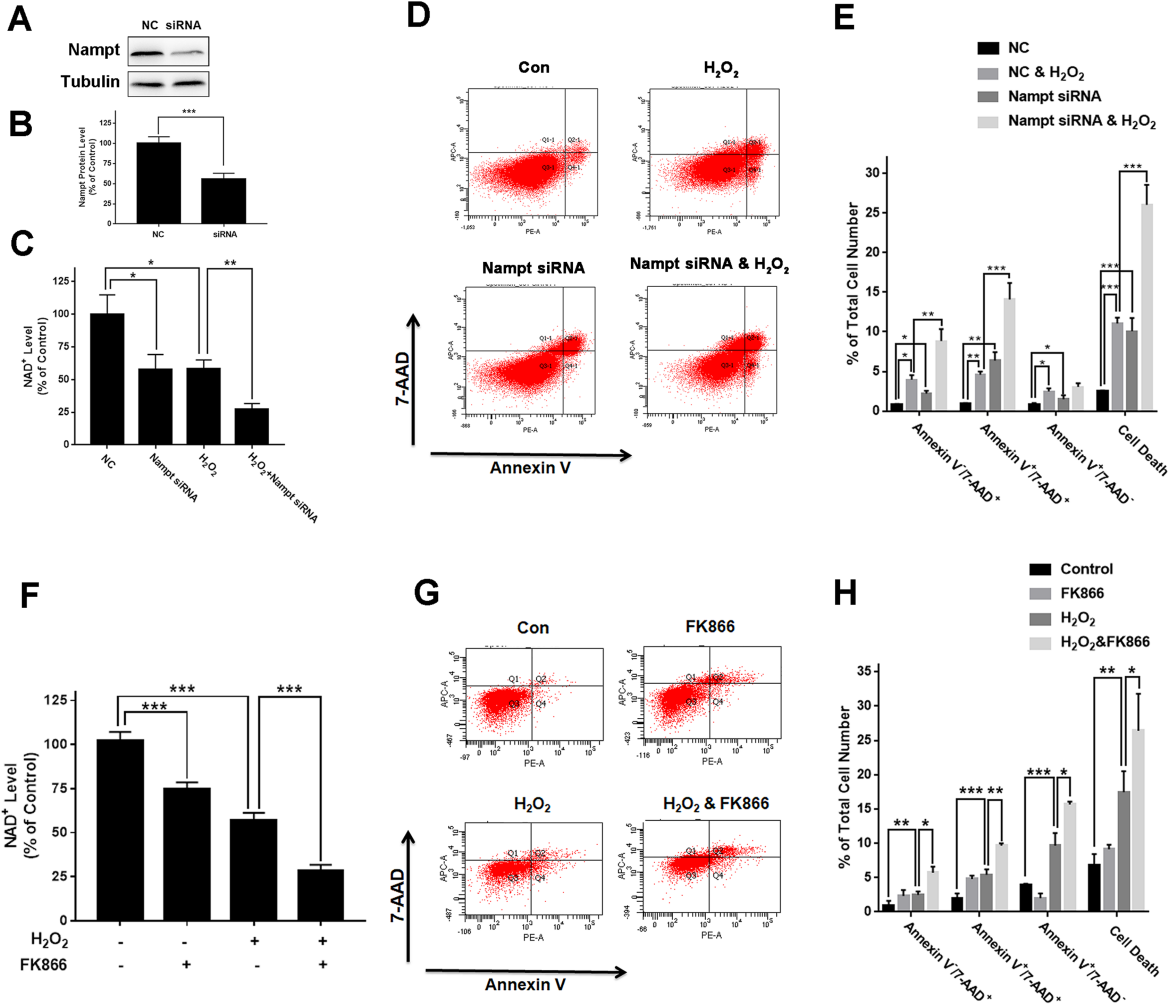
Nampt siRNA and FK866 significantly exacerbated H_2_O_2_-produced decreases in the intracellular NAD^+^ levels and cell survival of differentiated PC12 cells. ****(A, B) Treatment of the cells with Nampt siRNA led to a significant decrease in the Nampt protein level. PC12 cells were transfected with control or Nampt siRNA sequences for 48 h. There were 12 samples in the experiments. (C) Nampt siRNA treatment significantly exacerbated H_2_O_2_-produced decrease in the NAD^+^ levels of PC12 cells. (D, E) Nampt siRNA significantly exacerbated H_2_O_2_-produced late-stage apoptosis (Annexin V^+^/7-ADD^+^) and necrosis (Annexin V^-^/7-ADD^+^) of the cells. PC12 cells were transfected with control or Nampt siRNA sequences for 24 h, and then co-treated with 0.3 mM H_2_O_2_ for 6 h (for NAD^+^ assay) or 24 h (for flow cytometry assay). The data were pooled from three independent experiments. *N* = 3. There were three, three and three samples in these three independent experiments. *P < 0.05; **P < 0.01; ***P < 0.001. (F) FK866 significantly exacerbated H_2_O_2_-produced decrease in the NAD^+^ levels of PC12 cells. (G, H) FK866 significantly exacerbated H_2_O_2_-produced early-stage apoptosis (Annexin V^+^/7-ADD^−^), late-stage apoptosis (Annexin V^+^/7-ADD^+^) and necrosis (Annexin V^−^/7-ADD^+^) of the cells. PC12 cells were pre-treated with 10 nM FK866 for 30 min, and then co-treated with 0.3 mM H_2_O_2_ for 6 h (for NAD^+^ assay) or 23.5 h (for flow cytometry assay). The data were pooled from three independent experiments. *N* = 3. There were 3, 3 and 3 samples in these three independent experiments. *P < 0.05; **P < 0.01; ***P < 0.001.

To investigate the biological consequences of the H_2_O_2_-produced decreases in the Nampt levels of PC12 cells, we applied both Nampt siRNA and FK866 to determine whether the decreased Nampt affect the intracellular NAD^+^ levels and cell survival of the PC12 cells exposed to H_2_O_2_. The results showed that Nampt siRNA treatment led to a marked decrease in the Nampt level (Figs. 3A and 3B). Nampt siRNA treatment also significantly reduced the intracellular NAD^+^ level of PC12 cells under basal conditions (Fig. 3C). Moreover, the Nampt siRNA treatment further reduced the intracellular NAD^+^ level that was decreased by H_2_O_2_ treatment (Fig. 3C). Our results also showed that the Nampt siRNA treatment produced increased cell death under basal conditions (Figs. 3D and 3E). H_2_O_2_ treatment also increased the cell death, which was further enhanced by the Nampt siRNA treatment (Figs. 3D and 3E).

We found that FK866 further reduced the intracellular NAD^+^ level that was decreased by H_2_O_2_ treatment (Fig. 3F). Moreover, the FK866 treatment further enhanced the levels of the H_2_O_2_-produced necrosis, late-stage apoptosis and early-stage apoptosis (Figs. 3G and 3H).

### Nampt activator P7C3 suppressed the H_2_O_2_-produced decreases in the NAD^+^ levels and cell survival of PC12 cells

**Figure 4:**
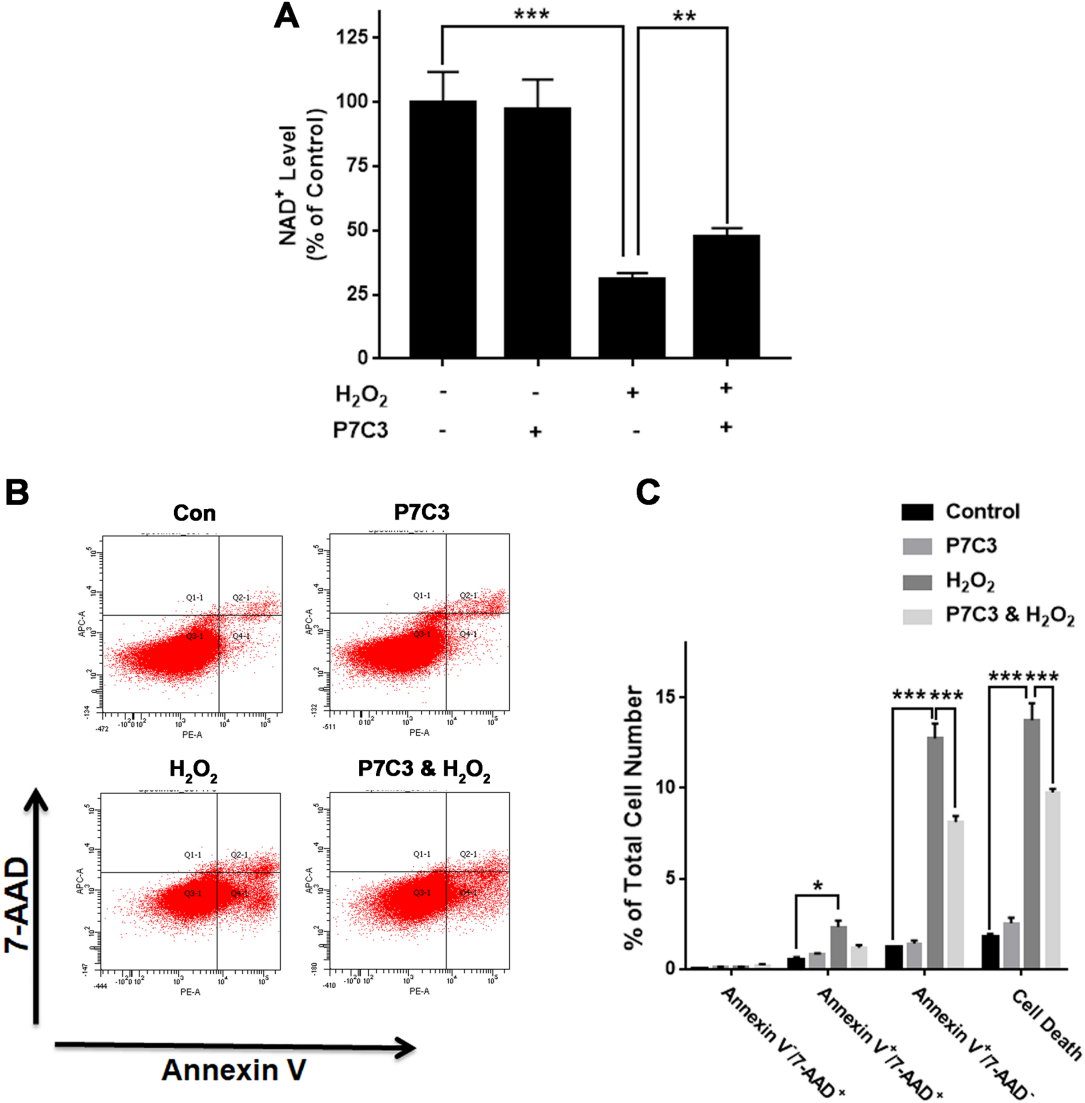
Nampt activator P7C3 significantly attenuated H_2_O_2_-produced decreases in the intracellular NAD^+^ levels and cell survival of differentiated PC12 cells. (A) P7C3 significantly attenuated the H_2_O_2_-produced decrease in the intracellular NAD^+^ levels of PC12 cells. (B, C) Nampt activator P7C3 significantly attenuated the H_2_O_2_-produced early-stage apoptosis (Annexin V^+^/7-ADD^−^) of the cells. PC12 cells were pre-treated with 10 mM P7C3 for 4 h, and then co-treated with 0.3 mM H_2_O_2_ for 6 h (for NAD^+^ assay) or 20 h (for flow cytometry assay). The data were pooled from three independent experiments. *N* = 3. There were three, three and three samples in these three independent experiments. *P < 0.05; **P < 0.01; ***P < 0.001.

To further investigate the biological consequences of the H_2_O_2_-produced reduction of the Nampt protein levels of PC12 cells, we used Nampt activator P7C3 to determine the roles of Nampt activation in the intracellular NAD^+^ levels and survival of PC12 cells exposed to H_2_O_2_: P7C3 attenuated the H_2_O_2_-produced decrease in the intracellular NAD^+^ level of PC12 cells (Fig. 4A). P7C3 also reduced the H_2_O_2_-produced increase in the cell death (Figs. 4B and 4C).

We also determined the effects of nicotinamide (NAM)—a substrate of Nampt—on both intracellular NAD^+^ levels and cell death of H_2_O_2_-treated PC12 cells. NAM attenuated the H_2_O_2_-produced decrease in the intracellular NAD^+^ level (Fig. S2A). NAM also attenuated the H_2_O_2_-produced increase in the cell death (Figs. S2B and S2C).

## Discussion

Our study has generated the following important findings: first, Nampt plays critical roles in both NAD^+^ synthesis and survival of PC12 cells under basal conditions; second, H_2_O_2_ produced significant decreases in both Nampt mRNA levels and Nampt protein levels of PC12 cells; and third, H_2_O_2_ induced cell death partially by producing the decreases in both Nampt mRNA levels and Nampt protein levels, since the Nampt siRNA and Nampt inhibitor significantly exacerbated the H_2_O_2_-produced decreases in the intracellular NAD^+^ level and cell survival, while the Nampt activator attenuated the H_2_O_2_-produced decreases in the intracellular NAD^+^ level and cell survival.

Cumulating evidence has suggested crucial roles of impaired NAD^+^ metabolism in both aging and multiple diseases (Ying, 2008; Ying et al., 2007; Zhang & Ying, 2019), indicating that it is necessary to determine the roles of NAD^+^ deficiency under these conditions (Ying, 2008; Ying et al., 2007; Zhang & Ying, 2019). In the tissues or organs under certain pathological conditions, including the metabolic organs that are exposed to HFD (Yoshino et al., 2011), Drosophila pink1 mutants (Lehmann, Loh & Martins, 2017), and aging (Jokinen et al., 2017; Yoshino et al., 2011), decreased levels of Nampt have been reported. However, there is significant deficiency in the mechanisms underlying the Nampt alterations under pathological conditions.

Our current study has shown that H_2_O_2_ was capable of decreasing both protein levels and mRNA levels of Nampt in PC12 cells. Increased oxidative stress has been found in aging and multiple pathological conditions (Rani et al., 2016; Ureshino et al., 2014; Zuo et al., 2019). Therefore, our finding has suggested a novel mechanism regarding the Nampt decrease in aging and under multiple pathological conditions. Due to the crucial roles of NAD^+^ deficiency in aging and numerous diseases, our finding has also suggested a novel relationship between the pathological alterations and NAD^+^ metabolism in aging and the diseases: The oxidative stress in the diseases and aging led to decreased Nampt, producing decreased NAD^+^ synthesis thus resulting in multiple pathological changes.

Our study found that oxidative stress produced biphasic changes of Nampt mRNA levels and Nampt protein levels, which is reasonable for the following reasons: Since NAD^+^ was involved in a variety of biological processes, oxidative stress-produced NAD^+^ decreases can lead to cell death. For defensing oxidative stress, both Nampt mRNA levels and Nampt protein levels can be induced by oxidative stress in the early phase. When the oxidative stress last for a significant duration of time, cells may lose the capacity to maintain the Nampt mRNA levels and Nampt protein levels, leading to reductions of the mRNA and protein levels of Nampt.

Our current findings have provided evidence that Nampt plays an important role in oxidative stress-induced cell death: Both the Nampt inhibitor and the Nampt siRNA significantly exacerbated the H_2_O_2_-produced reductions of the NAD^+^ level and cell survival, while the Nampt activator significantly attenuated the H_2_O_2_-induced reductions of the NAD^+^ level and cell survival. These observations have suggested a mechanism regarding oxidative stress-produced cell death at least for some cell types.

There are two NAD^+^ synthesis pathways in cells, while the effects of Nampt on the general NAD^+^-generating ability and survival of various cell types under basal conditions are unclear. Our current findings have indicated that Nampt plays critical roles in both NAD^+^ synthesis and cell survival under basal conditions in differentiated PC12 cells, since the Nampt siRNA and Nampt inhibitor FK866 produced profound decreases in both NAD^+^ levels and survival of the cells under basal conditions.

## Conclusion

Our study has indicated that oxidative stress is capable of producing decreased Nampt. Our study has also suggested that Nampt plays important roles in maintaining both intracellular NAD^+^ levels and cell survival under basal conditions and oxidative stress conditions.

## Supplemental Information

10.7717/peerj.11401/supp-1 Supplemental Information 1 Raw data.

10.7717/peerj.11401/supp-2 Supplemental Information 2 The PC12 cells used in our study showed the cell morphology of differentiated PC12.

10.7717/peerj.11401/supp-3 Supplemental Information 3 NAD^+^ precursor NAM significantly attenuated H_2_O_2_-produced decreases in the intracellular NAD^+^ levels and cell survival of differentiated PC12 cells. (A) NAM significantly attenuated the H_2_O_2_-produced decrease in the NAD^+^ levels of PC12 cells. (B, C) NAM significantly attenuated the H_2_O_2_-produced cell death. PC12 cells were pre-treated with 1 mM NAM for 1 h, and then co-treated with 0.3 mM H_2_O_2_ for 6 h (for NAD^+^ assay) or 23 h (for flow cytometry assay). The data were pooled from three independent experiments. *N* = 3. There were 3, 3 and 3 samples in these three independent experiments. *P < 0.05; **P < 0.01; ***P < 0.001.

## Additional Information and Declarations

### Data Availability

The following information was supplied regarding data availability: The raw measurements are available in the Supplemental File.
