# Supplementary figures and images for: Oxidative stress induces cell death partially by decreasing both mRNA and protein levels of nicotinamide phosphoribosyltransferase in differentiated PC12 cells
Source: PeerJ. 2021 May 14;9:e11401. doi: 10.7717/peerj.11401 (PMC8127959; doi:10.7717/peerj.11401)

# FACSDiva Version 6.1.3

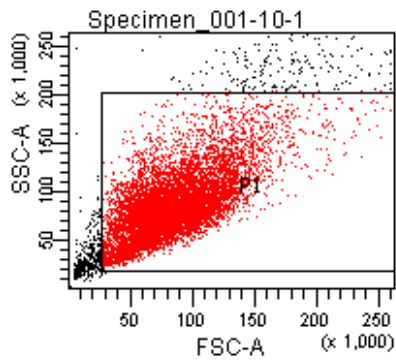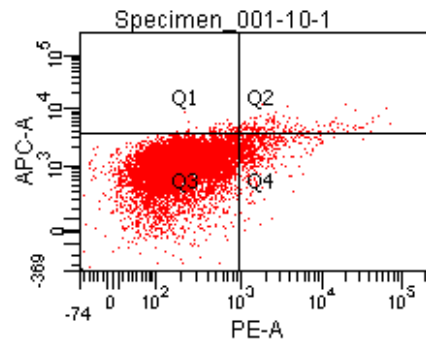

| Tube: 10-1   |         |         |        |
|--------------|---------|---------|--------|
| Population   | #Events | %Parent | %Total |
| ■ All Events | 10,000  | ####    | 100.0  |
| ■ P1         | 9,122   | 91.2    | 91.2   |
| ☒ Q1         | 53      | 0.6     | 0.5    |
| ☒ Q2         | 160     | 1.8     | 1.6    |
| ☒ Q3         | 8,313   | 91.1    | 83.1   |
| ☒ Q4         | 596     | 6.5     | 6.0    |

Supplement: Supplemental Information 1 [file peerj-09-11401-s001.zip › Raw data or code/Fig1B. FK866 Flow cytometry-based Annexin V7-AAD assay/10 nM FK866-1.pdf]

# FACSDiva Version 6.1.3

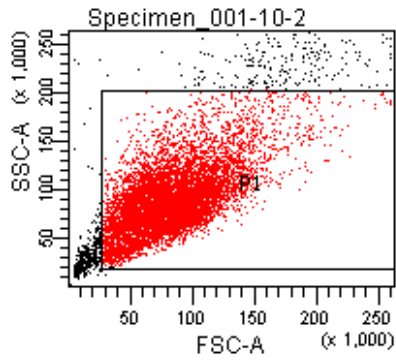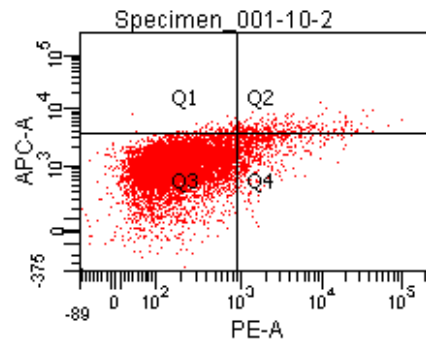

Tube: 10-2

Population

#Events

%Parent

%Total

■ All Events

10,000

####

100.0

■ P1

8,967

89.7

89.7

□ Q1

63

0.7

0.6

□ Q2

220

2.5

2.2

□ Q3

8,140

90.8

81.4

□ Q4

544

6.1

5.4

Supplement: Supplemental Information 1 [file peerj-09-11401-s001.zip › Raw data or code/Fig1B. FK866 Flow cytometry-based Annexin V7-AAD assay/10 nM FK866-2.pdf]

# FACSDiva Version 6.1.3

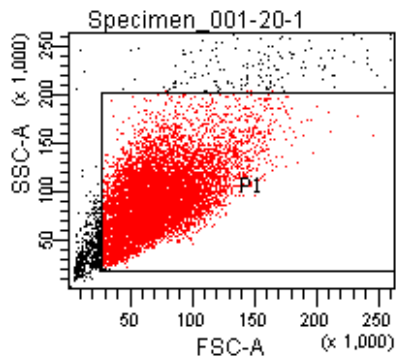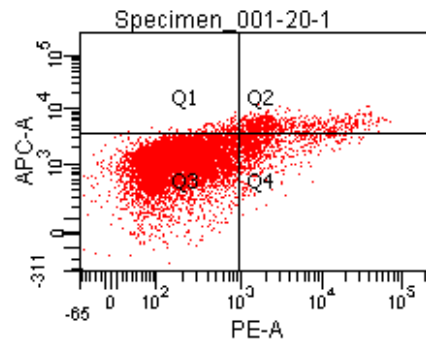

| Tube: 20-1   |         |         |        |
|--------------|---------|---------|--------|
| Population   | #Events | %Parent | %Total |
| ■ All Events | 10,000  | ####    | 100.0  |
| ■ P1         | 9,171   | 91.7    | 91.7   |
| ☒ Q1         | 108     | 1.2     | 1.1    |
| ☒ Q2         | 671     | 7.3     | 6.7    |
| ☒ Q3         | 7,602   | 82.9    | 76.0   |
| ☒ Q4         | 790     | 8.6     | 7.9    |

Supplement: Supplemental Information 1 [file peerj-09-11401-s001.zip › Raw data or code/Fig1B. FK866 Flow cytometry-based Annexin V7-AAD assay/20 nM FK866-1.pdf]

# FACSDiva Version 6.1.3

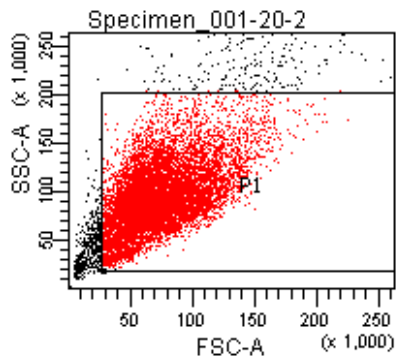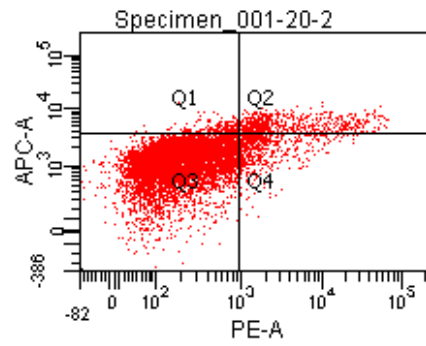

| Tube: 20-2   |         |         |        |
|--------------|---------|---------|--------|
| Population   | #Events | %Parent | %Total |
| ■ All Events | 10,000  | ####    | 100.0  |
| ■ P1         | 9,226   | 92.3    | 92.3   |
| ☒ Q1         | 219     | 2.4     | 2.2    |
| ☒ Q2         | 701     | 7.6     | 7.0    |
| ☒ Q3         | 7,638   | 82.8    | 76.4   |
| ☒ Q4         | 668     | 7.2     | 6.7    |

Supplement: Supplemental Information 1 [file peerj-09-11401-s001.zip › Raw data or code/Fig1B. FK866 Flow cytometry-based Annexin V7-AAD assay/20 nM FK866-2.pdf]

# FACSDiva Version 6.1.3

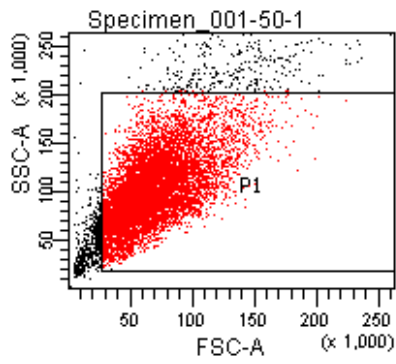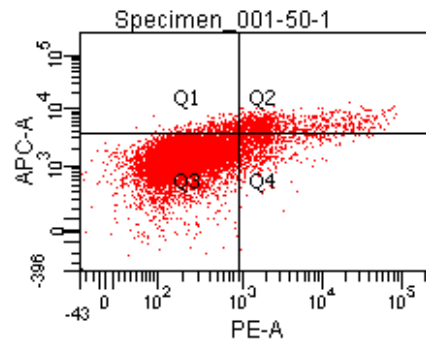

| Tube: 50-1   |         |         |        |
|--------------|---------|---------|--------|
| Population   | #Events | %Parent | %Total |
| ■ All Events | 10,000  | ####    | 100.0  |
| ■ P1         | 8,927   | 89.3    | 89.3   |
| ☒ Q1         | 288     | 3.2     | 2.9    |
| ☒ Q2         | 862     | 9.7     | 8.6    |
| ☒ Q3         | 7,036   | 78.8    | 70.4   |
| ☒ Q4         | 741     | 8.3     | 7.4    |

Supplement: Supplemental Information 1 [file peerj-09-11401-s001.zip › Raw data or code/Fig1B. FK866 Flow cytometry-based Annexin V7-AAD assay/50 nM FK866-1.pdf]

# FACSDiva Version 6.1.3

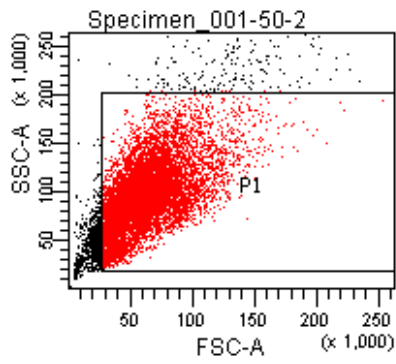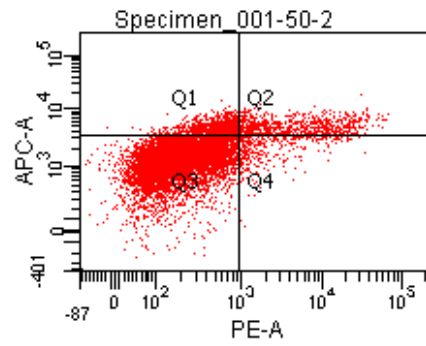

| Tube: 50-2   |         |         |        |
|--------------|---------|---------|--------|
| Population   | #Events | %Parent | %Total |
| ■ All Events | 10,000  | ####    | 100.0  |
| ■ P1         | 8,922   | 89.2    | 89.2   |
| ☒ Q1         | 749     | 8.4     | 7.5    |
| ☒ Q2         | 713     | 8.0     | 7.1    |
| ☒ Q3         | 6,961   | 78.0    | 69.6   |
| ☒ Q4         | 499     | 5.6     | 5.0    |

Supplement: Supplemental Information 1 [file peerj-09-11401-s001.zip › Raw data or code/Fig1B. FK866 Flow cytometry-based Annexin V7-AAD assay/50 nM FK866-2.pdf]

# FACSDiva Version 6.1.3

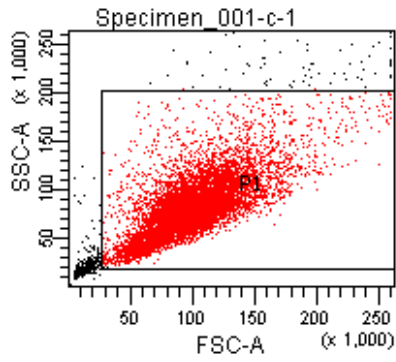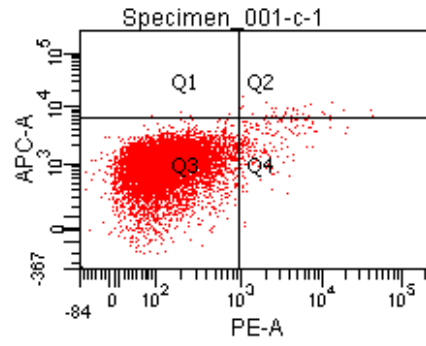

| Tube: c-1    |         |         |        |
|--------------|---------|---------|--------|
| Population   | #Events | %Parent | %Total |
| ■ All Events | 10,000  | ####    | 100.0  |
| ■ P1         | 9,301   | 93.0    | 93.0   |
| □ Q1         | 5       | 0.1     | 0.0    |
| □ Q2         | 34      | 0.4     | 0.3    |
| □ Q3         | 9,061   | 97.4    | 90.6   |
| □ Q4         | 201     | 2.2     | 2.0    |

Supplement: Supplemental Information 1 [file peerj-09-11401-s001.zip › Raw data or code/Fig1B. FK866 Flow cytometry-based Annexin V7-AAD assay/Control-1.pdf]

# FACSDiva Version 6.1.3

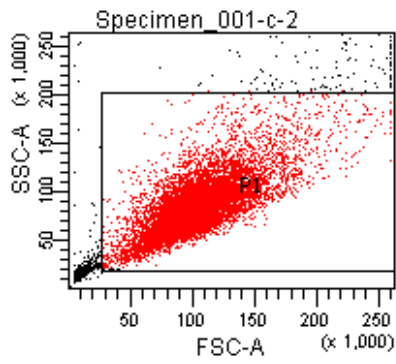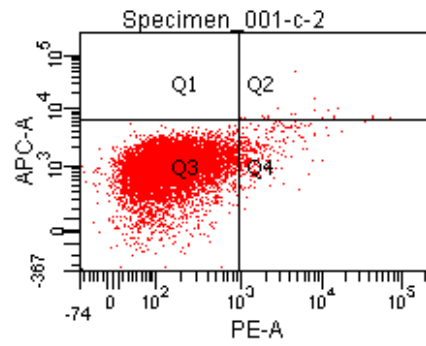

| Tube: c-2    |         |         |        |
|--------------|---------|---------|--------|
| Population   | #Events | %Parent | %Total |
| ■ All Events | 10,000  | ####    | 100.0  |
| ■ P1         | 9,323   | 93.2    | 93.2   |
| ☒ Q1         | 1       | 0.0     | 0.0    |
| ☒ Q2         | 21      | 0.2     | 0.2    |
| ☒ Q3         | 9,020   | 96.7    | 90.2   |
| ☒ Q4         | 281     | 3.0     | 2.8    |

Supplement: Supplemental Information 1 [file peerj-09-11401-s001.zip › Raw data or code/Fig1B. FK866 Flow cytometry-based Annexin V7-AAD assay/Control-2.pdf]

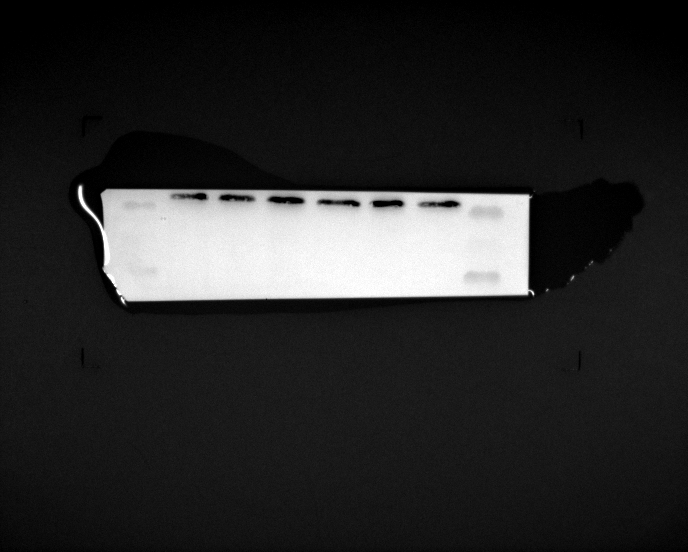

Supplement: Supplemental Information 1 [file peerj-09-11401-s001.zip › Raw data or code/Fig2B,C,D. NAMPT Western blot/Tubulin 12h-10s merge.tif]

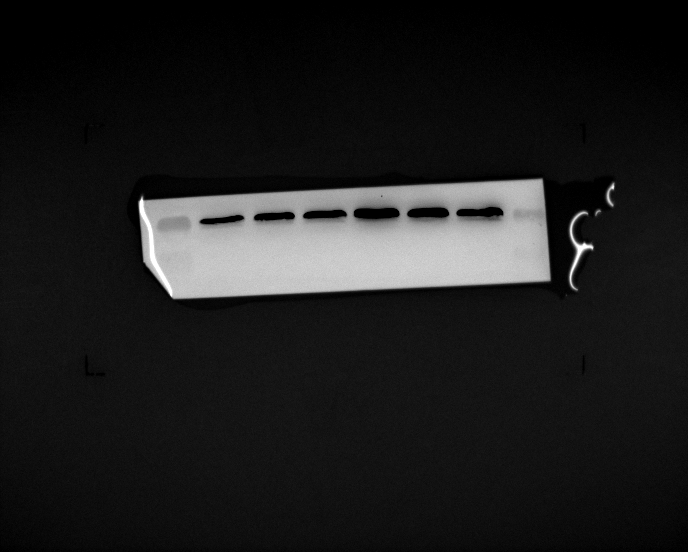

Supplement: Supplemental Information 1 [file peerj-09-11401-s001.zip › Raw data or code/Fig2B,C,D. NAMPT Western blot/Tubulin 24h-10s merge.tif]

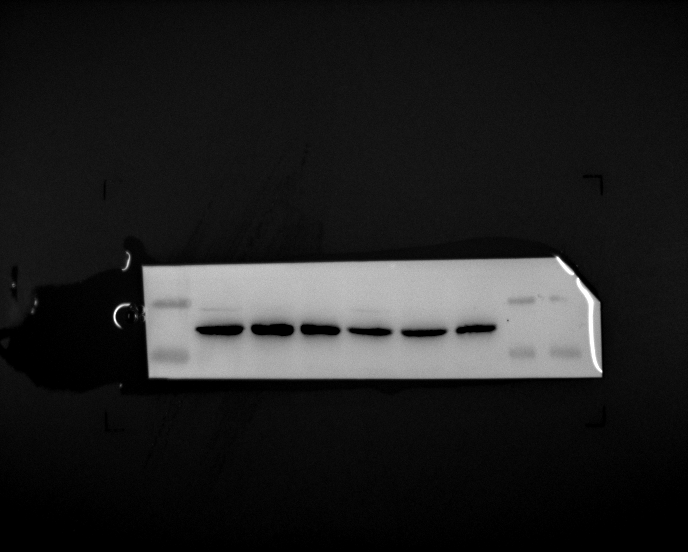

Supplement: Supplemental Information 1 [file peerj-09-11401-s001.zip › Raw data or code/Fig2B,C,D. NAMPT Western blot/Tubulin 48h-10s merge.tif]

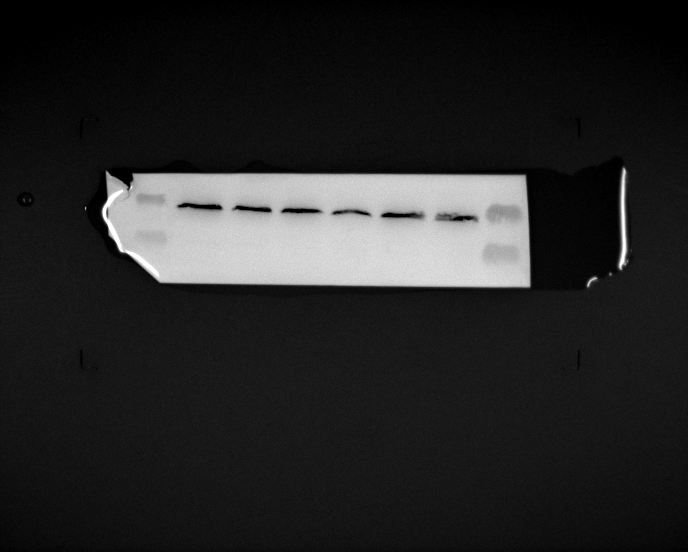

Supplement: Supplemental Information 1 [file peerj-09-11401-s001.zip › Raw data or code/Fig2B,C,D. NAMPT Western blot/nampt 12h-20s merge.tif]

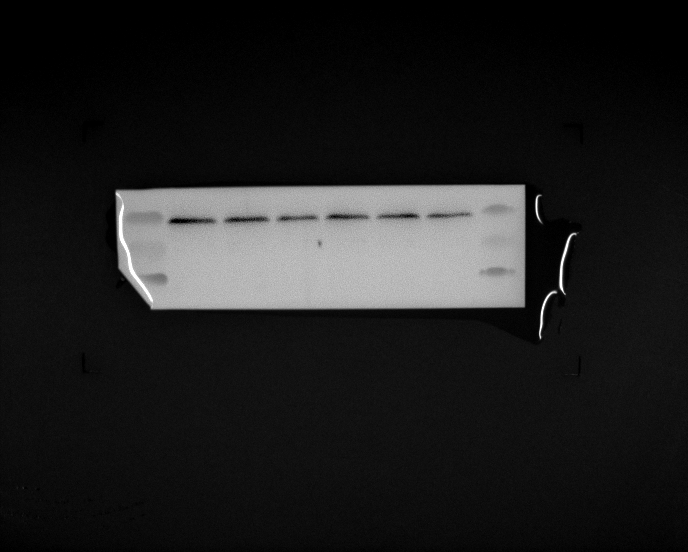

Supplement: Supplemental Information 1 [file peerj-09-11401-s001.zip › Raw data or code/Fig2B,C,D. NAMPT Western blot/nampt 24h-10s merge.tif]

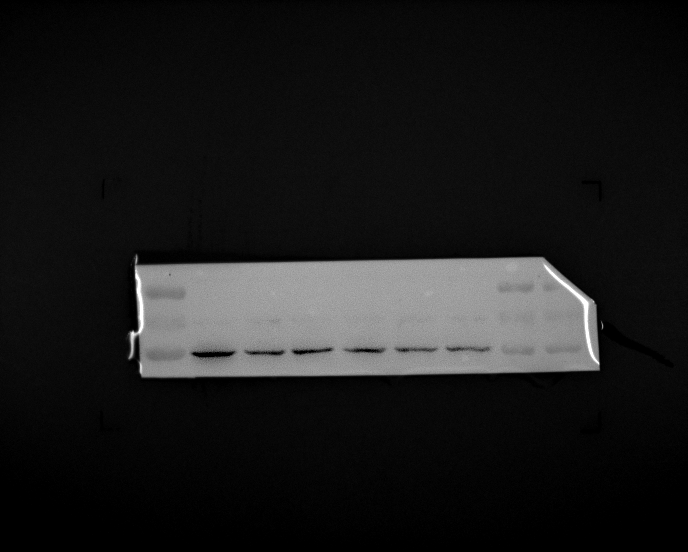

Supplement: Supplemental Information 1 [file peerj-09-11401-s001.zip › Raw data or code/Fig2B,C,D. NAMPT Western blot/nampt 48h-20s merge.tif]

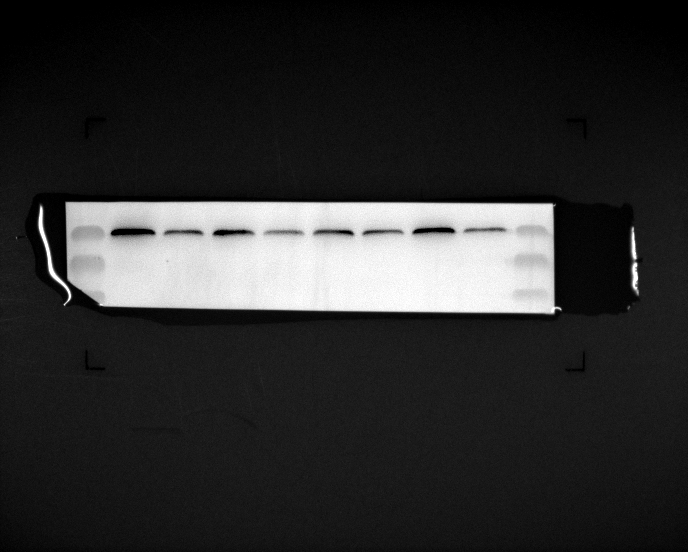

Supplement: Supplemental Information 1 [file peerj-09-11401-s001.zip › Raw data or code/Fig3A. NAMPT siRNA/NAMPT siRNA 10S MERGE.tif]

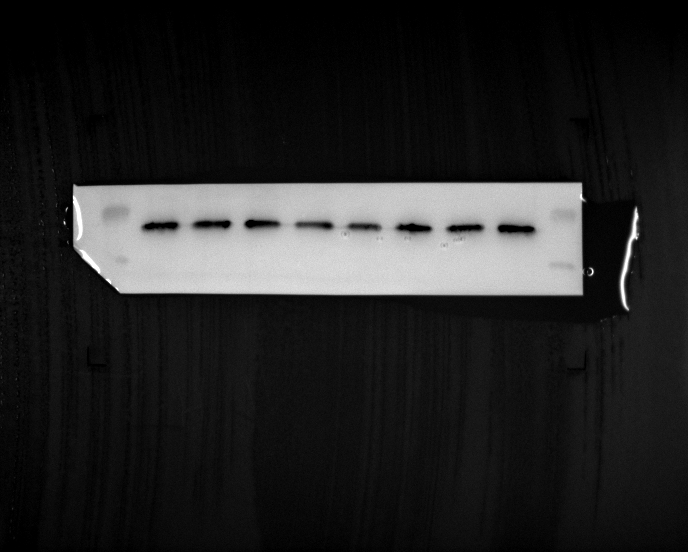

Supplement: Supplemental Information 1 [file peerj-09-11401-s001.zip › Raw data or code/Fig3A. NAMPT siRNA/Tubulin 10S MERGE.tif]

# FACSDiva Version 6.1.3

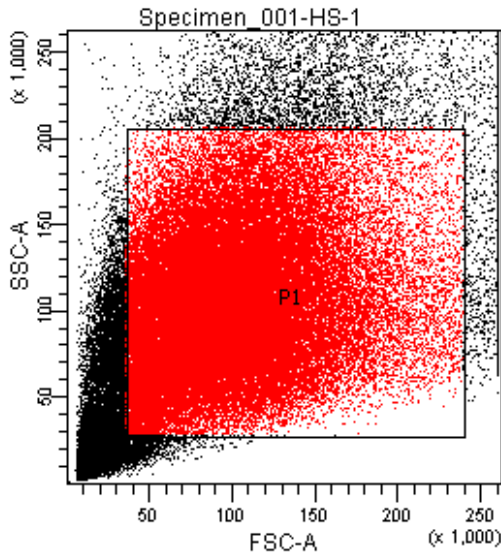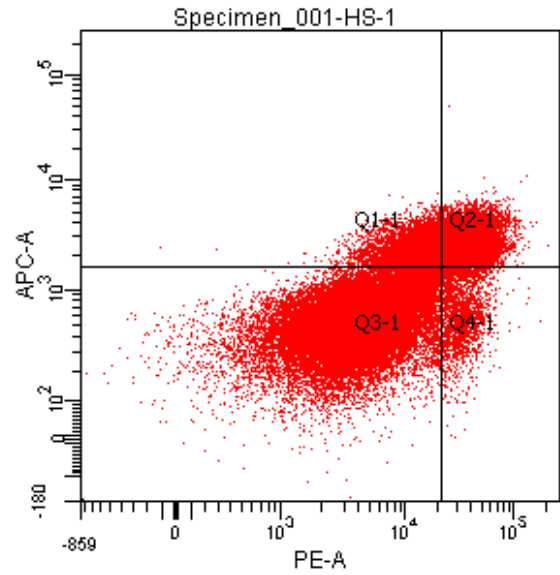

| Tube: HS-1   |         |         |        |
|--------------|---------|---------|--------|
| Population   | #Events | %Parent | %Total |
| ■ All Events | 100,000 | ###     | 100.0  |
| ■ P1         | 69,879  | 69.9    | 69.9   |
| ☒ Q1-1       | 6,163   | 8.8     | 6.2    |
| ☒ Q2-1       | 17,107  | 24.5    | 17.1   |
| ☒ Q3-1       | 43,851  | 62.8    | 43.9   |
| ☒ Q4-1       | 2,758   | 3.9     | 2.8    |

Supplement: Supplemental Information 1 [file peerj-09-11401-s001.zip › Raw data or code/Fig3C. NAMPT siRNA &H2O2 Flow cytometry-based Annexin V7-AAD assay/H2O2&NAMPT siRNA.pdf]

# FACSDiva Version 6.1.3

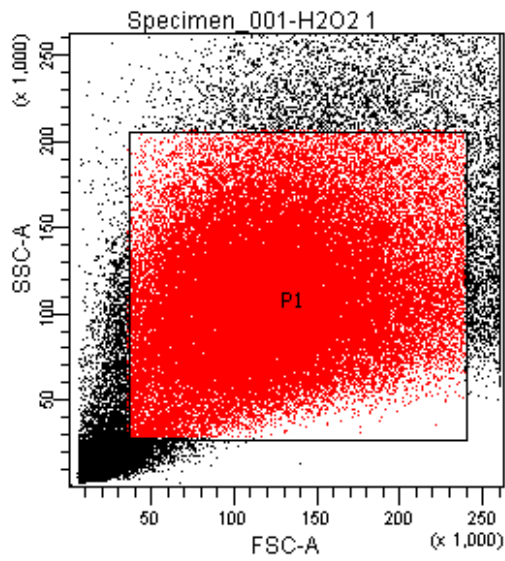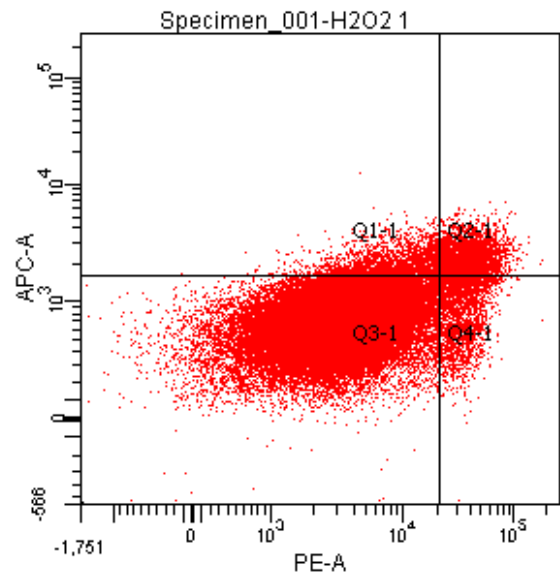

| Tube: H2O2 1 |         |         |        |
|--------------|---------|---------|--------|
| Population   | #Events | %Parent | %Total |
| ■ All Events | 100,000 | ###     | 100.0  |
| ■ P1         | 71,102  | 71.1    | 71.1   |
| ☒ Q1-1       | 2,098   | 3.0     | 2.1    |
| ☒ Q2-1       | 3,463   | 4.9     | 3.5    |
| ☒ Q3-1       | 62,622  | 88.1    | 62.6   |
| ☒ Q4-1       | 2,919   | 4.1     | 2.9    |

Supplement: Supplemental Information 1 [file peerj-09-11401-s001.zip › Raw data or code/Fig3C. NAMPT siRNA &H2O2 Flow cytometry-based Annexin V7-AAD assay/H2O2.pdf]

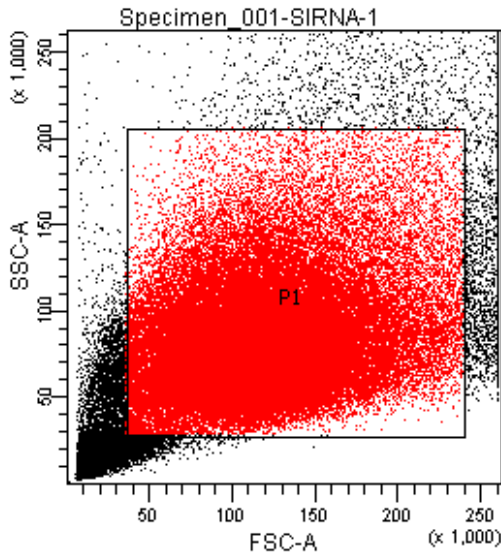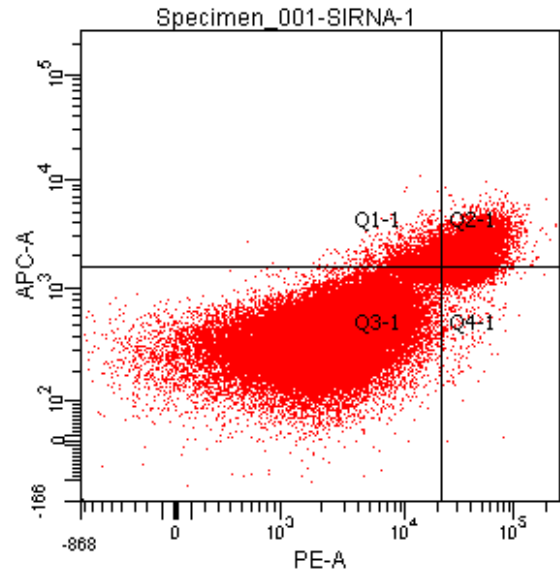

| Tube: SIRNA-1 |         |         |        |
|---------------|---------|---------|--------|
| Population    | #Events | %Parent | %Total |
| ■ All Events  | 100,000 | ###     | 100.0  |
| ■ P1          | 73,453  | 73.5    | 73.5   |
| ☒ Q1-1        | 1,248   | 1.7     | 1.2    |
| ☒ Q2-1        | 7,445   | 10.1    | 7.4    |
| ☒ Q3-1        | 61,493  | 83.7    | 61.5   |
| ☒ Q4-1        | 3,267   | 4.4     | 3.3    |

Supplement: Supplemental Information 1 [file peerj-09-11401-s001.zip › Raw data or code/Fig3C. NAMPT siRNA &H2O2 Flow cytometry-based Annexin V7-AAD assay/NAMPT siRNA.pdf]

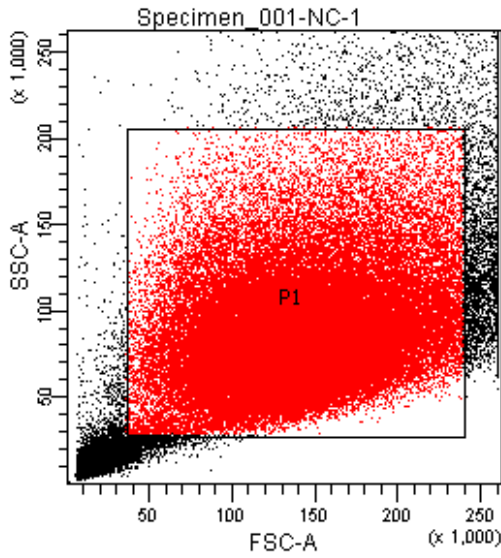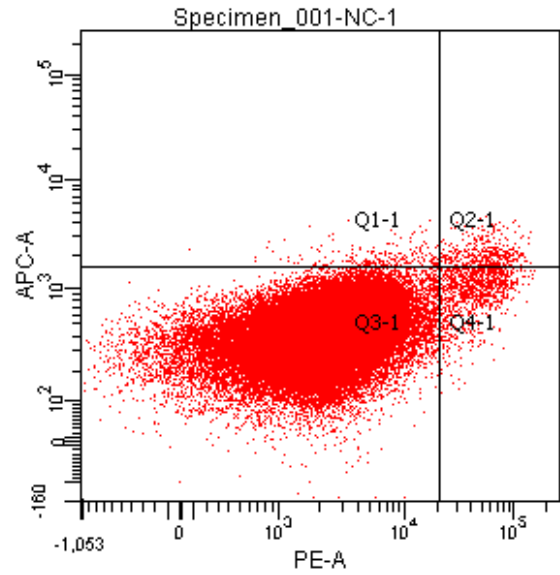

| Tube: NC-1   |         |         |        |
|--------------|---------|---------|--------|
| Population   | #Events | %Parent | %Total |
| ■ All Events | 100,000 | ###     | 100.0  |
| ■ P1         | 76,486  | 76.5    | 76.5   |
| ☒ Q1-1       | 180     | 0.2     | 0.2    |
| ☒ Q2-1       | 344     | 0.4     | 0.3    |
| ☒ Q3-1       | 74,857  | 97.9    | 74.9   |
| ☒ Q4-1       | 1,105   | 1.4     | 1.1    |

Supplement: Supplemental Information 1 [file peerj-09-11401-s001.zip › Raw data or code/Fig3C. NAMPT siRNA &H2O2 Flow cytometry-based Annexin V7-AAD assay/NC.pdf]

# FACSDiva Version 6.1.3

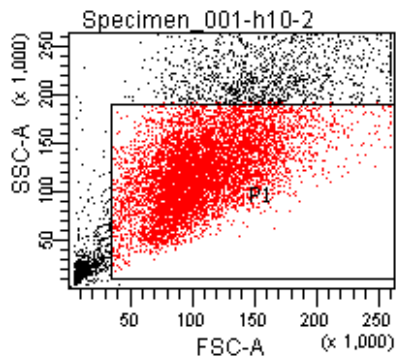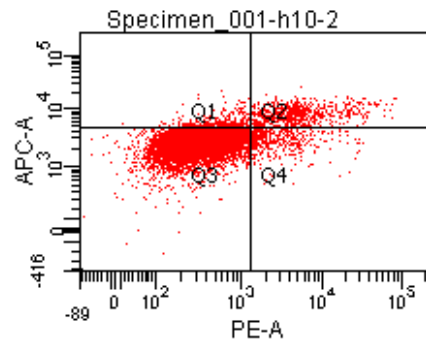

| Tube: h10-2  |         |         |        |
|--------------|---------|---------|--------|
| Population   | #Events | %Parent | %Total |
| ■ All Events | 10,000  | ####    | 100.0  |
| ■ P1         | 7,481   | 74.8    | 74.8   |
| ☒ Q1         | 361     | 4.8     | 3.6    |
| ☒ Q2         | 692     | 9.3     | 6.9    |
| ☒ Q3         | 5,975   | 79.9    | 59.8   |
| ☒ Q4         | 453     | 6.1     | 4.5    |

Supplement: Supplemental Information 1 [file peerj-09-11401-s001.zip › Raw data or code/Fig3F. FK866&H2O2 Flow cytometry-based Annexin V7-AAD assay/10 nM FK866 & H2O2.pdf]

# FACSDiva Version 6.1.3

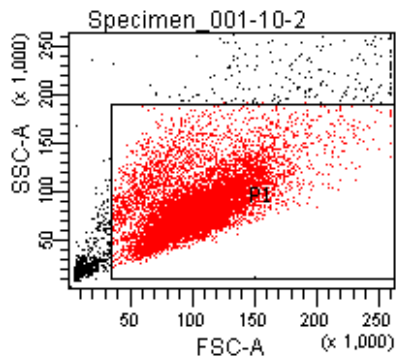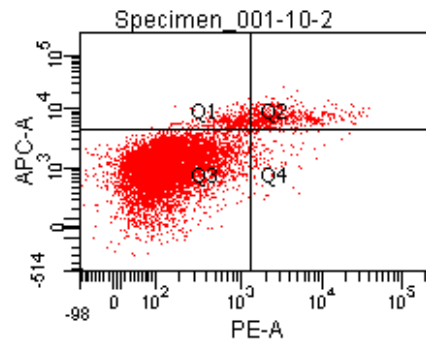

| Tube: 10-2   |         |         |        |
|--------------|---------|---------|--------|
| Population   | #Events | %Parent | %Total |
| ■ All Events | 10,000  | ###     | 100.0  |
| ■ P1         | 8,985   | 89.8    | 89.8   |
| ☒ Q1         | 284     | 3.2     | 2.8    |
| ☒ Q2         | 479     | 5.3     | 4.8    |
| ☒ Q3         | 8,108   | 90.2    | 81.1   |
| ☒ Q4         | 114     | 1.3     | 1.1    |

Supplement: Supplemental Information 1 [file peerj-09-11401-s001.zip › Raw data or code/Fig3F. FK866&H2O2 Flow cytometry-based Annexin V7-AAD assay/10 nM FK866.pdf]

# FACSDiva Version 6.1.3

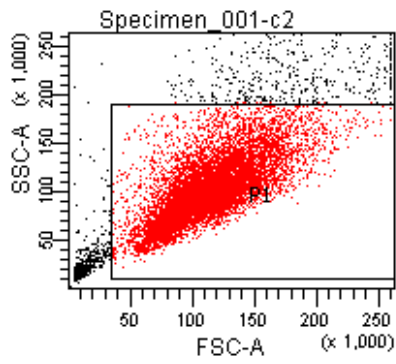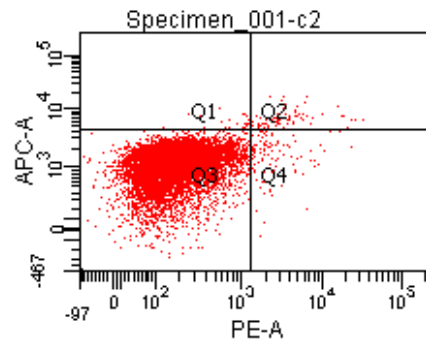

| Tube: c2     |         |         |        |
|--------------|---------|---------|--------|
| Population   | #Events | %Parent | %Total |
| ■ All Events | 10,000  | ####    | 100.0  |
| ■ P1         | 8,807   | 88.1    | 88.1   |
| ☒ Q1         | 23      | 0.3     | 0.2    |
| ☒ Q2         | 69      | 0.8     | 0.7    |
| ☒ Q3         | 8,591   | 97.5    | 85.9   |
| ☒ Q4         | 124     | 1.4     | 1.2    |

Supplement: Supplemental Information 1 [file peerj-09-11401-s001.zip › Raw data or code/Fig3F. FK866&H2O2 Flow cytometry-based Annexin V7-AAD assay/Control.pdf]

# FACSDiva Version 6.1.3

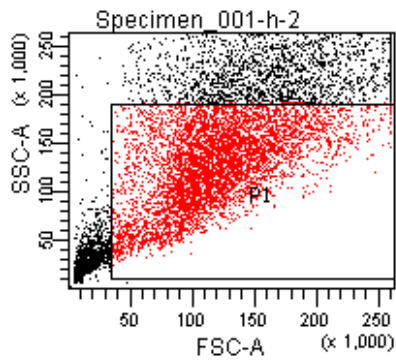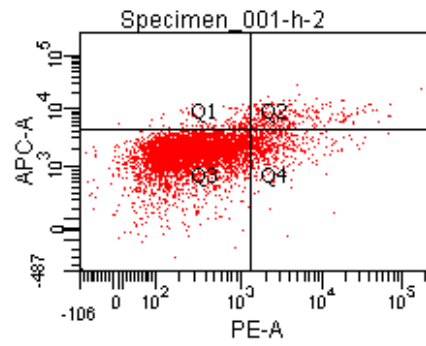

| Tube: h-2    |         |         |        |
|--------------|---------|---------|--------|
| Population   | #Events | %Parent | %Total |
| ■ All Events | 10,000  | ####    | 100.0  |
| ■ P1         | 5,369   | 53.7    | 53.7   |
| ☒ Q1         | 107     | 2.0     | 1.1    |
| ☒ Q2         | 248     | 4.6     | 2.5    |
| ☒ Q3         | 4,595   | 85.6    | 46.0   |
| ☒ Q4         | 419     | 7.8     | 4.2    |

Supplement: Supplemental Information 1 [file peerj-09-11401-s001.zip › Raw data or code/Fig3F. FK866&H2O2 Flow cytometry-based Annexin V7-AAD assay/H2O2.pdf]

# FACSDiva Version 6.1.3

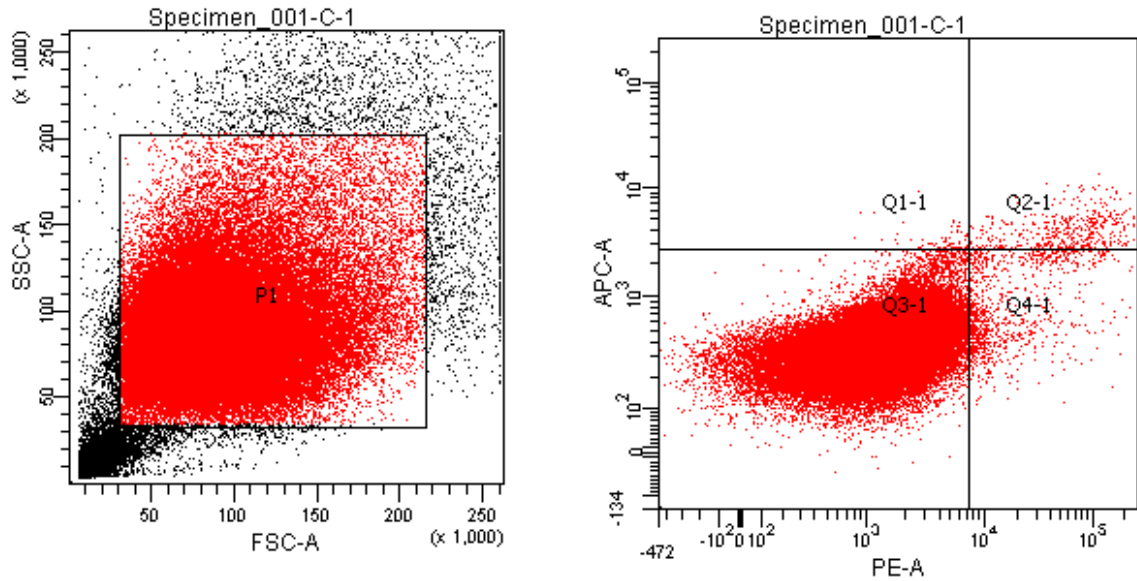

| Tube: C-1    |         |         |        |
|--------------|---------|---------|--------|
| Population   | #Events | %Parent | %Total |
| ■ All Events | 100,000 | ###     | 100.0  |
| ■ P1         | 84,945  | 84.9    | 84.9   |
| ☒ Q1-1       | 56      | 0.1     | 0.1    |
| ☒ Q2-1       | 415     | 0.5     | 0.4    |
| ☒ Q3-1       | 83,317  | 98.1    | 83.3   |
| ☒ Q4-1       | 1,157   | 1.4     | 1.2    |

Supplement: Supplemental Information 1 [file peerj-09-11401-s001.zip › Raw data or code/Fig4B. P7C3&H2O2 Flow cytometry-based Annexin V7-AAD assay/Control.pdf]

# FACSDiva Version 6.1.3

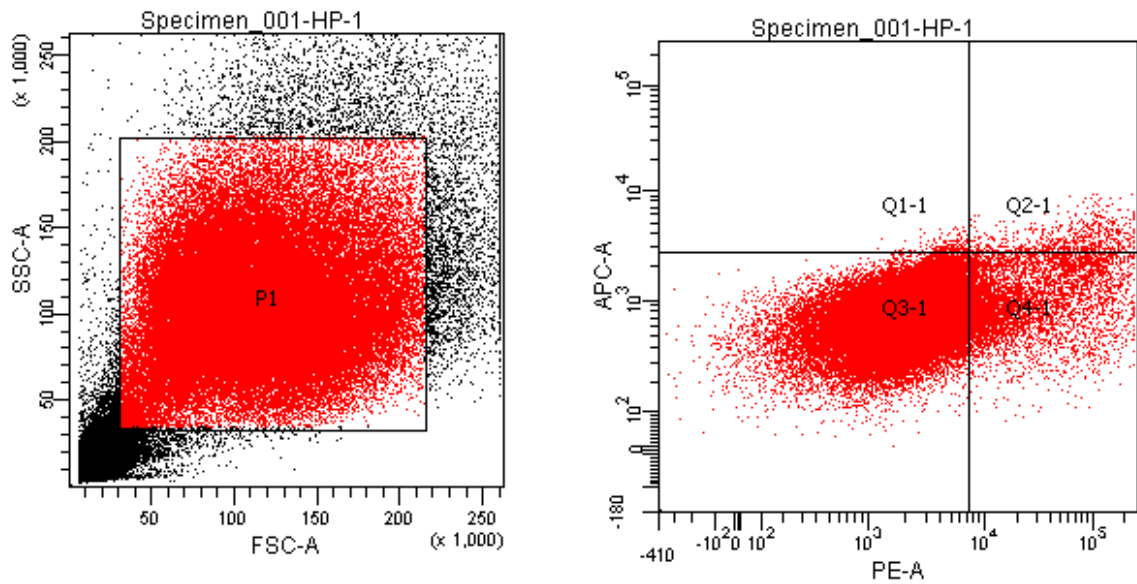

| Tube: HP-1   |         |         |        |
|--------------|---------|---------|--------|
| Population   | #Events | %Parent | %Total |
| ■ All Events | 100,000 | ###     | 100.0  |
| ■ P1         | 73,839  | 73.8    | 73.8   |
| ☒ Q1-1       | 153     | 0.2     | 0.2    |
| ☒ Q2-1       | 630     | 0.9     | 0.6    |
| ☒ Q3-1       | 66,928  | 90.6    | 66.9   |
| ☒ Q4-1       | 6,128   | 8.3     | 6.1    |

Supplement: Supplemental Information 1 [file peerj-09-11401-s001.zip › Raw data or code/Fig4B. P7C3&H2O2 Flow cytometry-based Annexin V7-AAD assay/H2O2&P7C3.pdf]

# FACSDiva Version 6.1.3

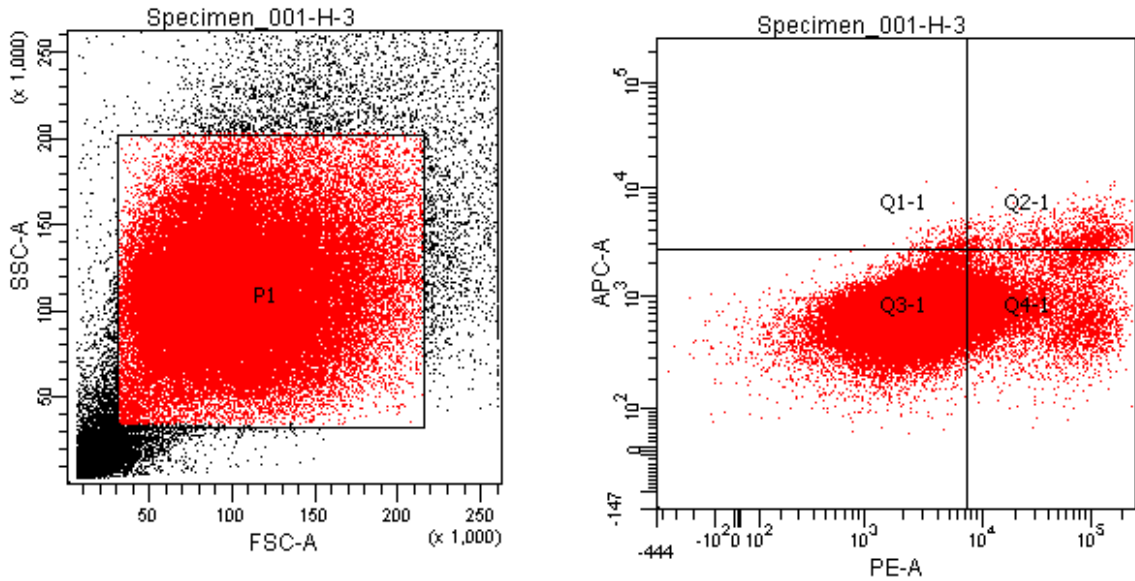

| Tube: H-3    |         |         |        |
|--------------|---------|---------|--------|
| Population   | #Events | %Parent | %Total |
| ■ All Events | 100,000 | ###     | 100.0  |
| ■ P1         | 78,249  | 78.2    | 78.2   |
| ☒ Q1-1       | 176     | 0.2     | 0.2    |
| ☒ Q2-1       | 1,161   | 1.5     | 1.2    |
| ☒ Q3-1       | 65,004  | 83.1    | 65.0   |
| ☒ Q4-1       | 11,908  | 15.2    | 11.9   |

Supplement: Supplemental Information 1 [file peerj-09-11401-s001.zip › Raw data or code/Fig4B. P7C3&H2O2 Flow cytometry-based Annexin V7-AAD assay/H2O2.pdf]

# FACSDiva Version 6.1.3

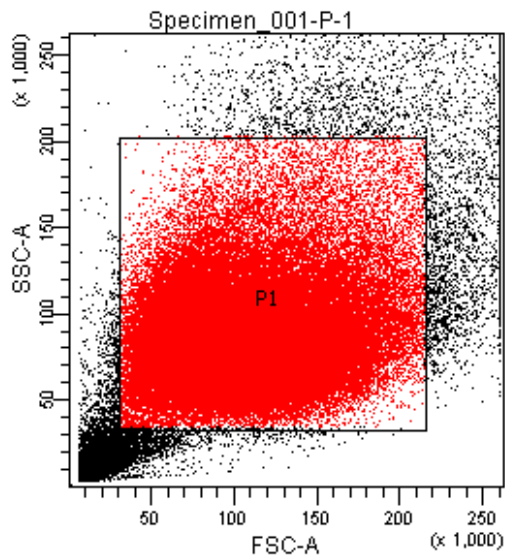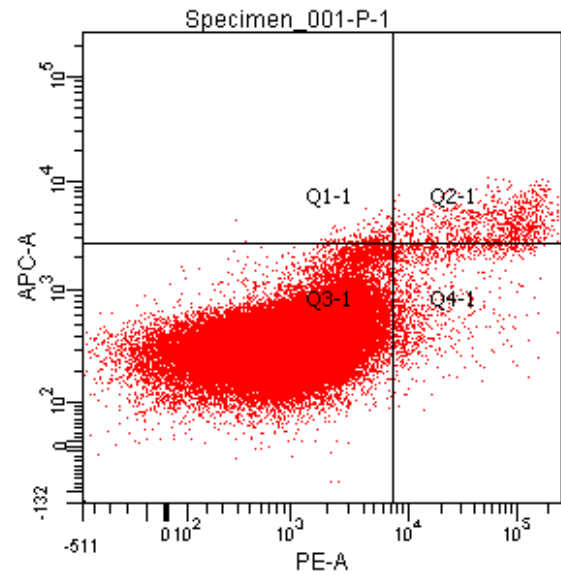

| Tube: P-1    |         |         |        |
|--------------|---------|---------|--------|
| Population   | #Events | %Parent | %Total |
| ■ All Events | 100,000 | ###     | 100.0  |
| ■ P1         | 82,408  | 82.4    | 82.4   |
| ☒ Q1-1       | 126     | 0.2     | 0.1    |
| ☒ Q2-1       | 809     | 1.0     | 0.8    |
| ☒ Q3-1       | 80,243  | 97.4    | 80.2   |
| ☒ Q4-1       | 1,230   | 1.5     | 1.2    |

Supplement: Supplemental Information 1 [file peerj-09-11401-s001.zip › Raw data or code/Fig4B. P7C3&H2O2 Flow cytometry-based Annexin V7-AAD assay/P7C3.pdf]

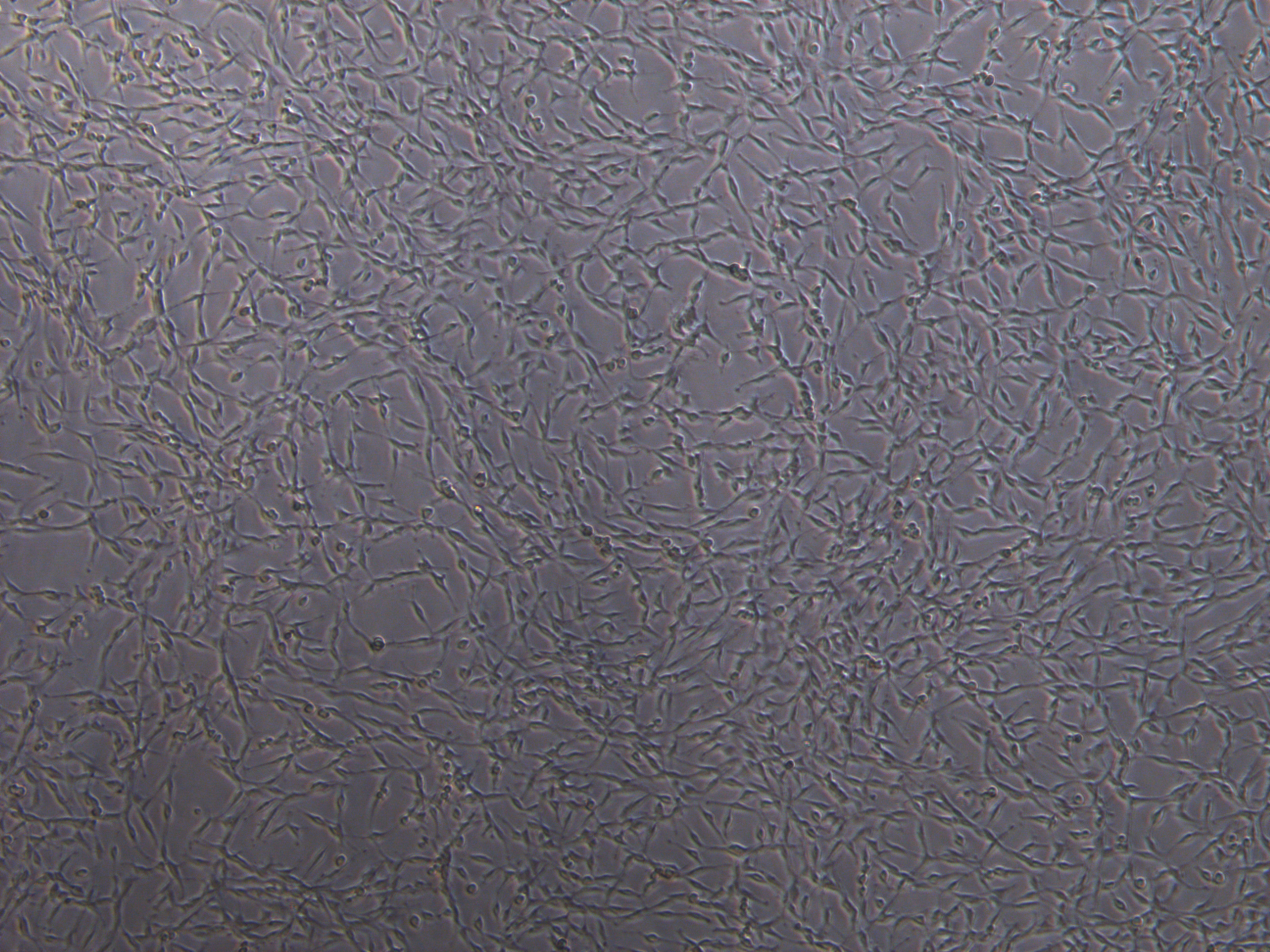

Supplement: Supplemental Information 1 [file peerj-09-11401-s001.zip › Raw data or code/s-Fig1.tif]

# FACSDiva Version 6.1.3

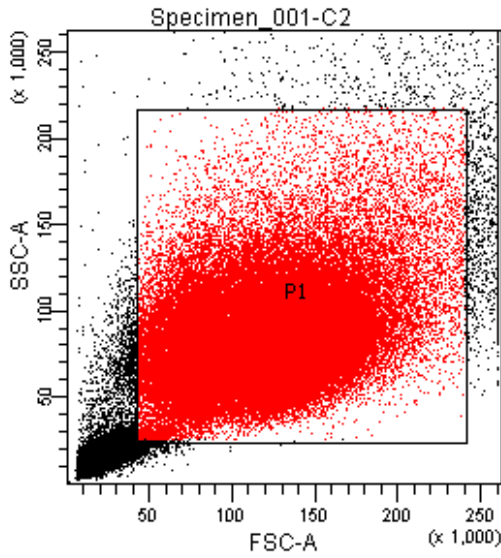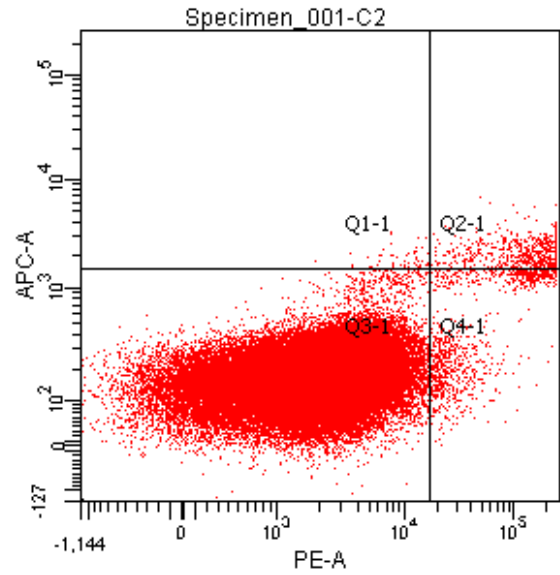

| Tube: C2     |         |         |        |
|--------------|---------|---------|--------|
| Population   | #Events | %Parent | %Total |
| ■ All Events | 100,000 | ###     | 100.0  |
| ■ P1         | 83,046  | 83.0    | 83.0   |
| ☒ Q1-1       | 50      | 0.1     | 0.0    |
| ☒ Q2-1       | 561     | 0.7     | 0.6    |
| ☒ Q3-1       | 81,455  | 98.1    | 81.5   |
| ☒ Q4-1       | 980     | 1.2     | 1.0    |

Supplement: Supplemental Information 1 [file peerj-09-11401-s001.zip › Raw data or code/s-Fig2B. NAM&H2O2 Flow cytometry-based Annexin V7-AAD assay/Control.pdf]

# FACSDiva Version 6.1.3

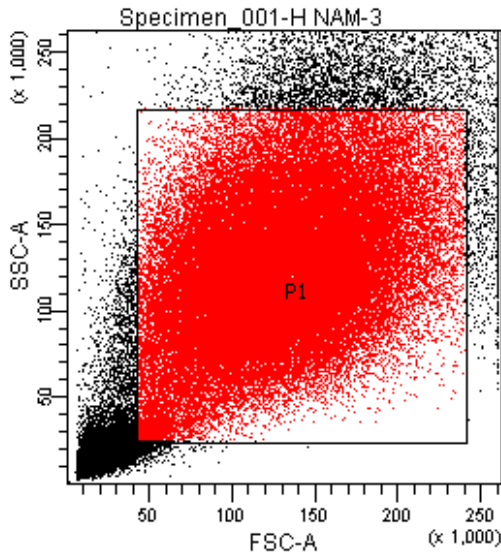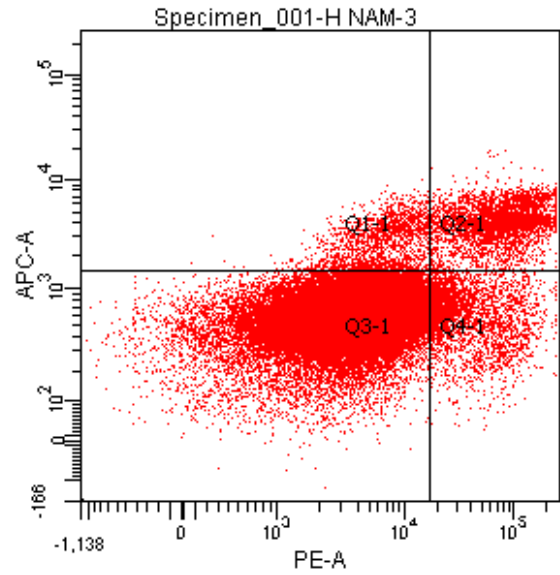

| Tube: H NAM-3 |         |         |        |
|---------------|---------|---------|--------|
| Population    | #Events | %Parent | %Total |
| ■ All Events  | 100,000 | ###     | 100.0  |
| ■ P1          | 74,372  | 74.4    | 74.4   |
| ☒ Q1-1        | 1,658   | 2.2     | 1.7    |
| ☒ Q2-1        | 3,667   | 4.9     | 3.7    |
| ☒ Q3-1        | 65,533  | 88.1    | 65.5   |
| ☒ Q4-1        | 3,514   | 4.7     | 3.5    |

Supplement: Supplemental Information 1 [file peerj-09-11401-s001.zip › Raw data or code/s-Fig2B. NAM&H2O2 Flow cytometry-based Annexin V7-AAD assay/H2O2& NAM.pdf]

# FACSDiva Version 6.1.3

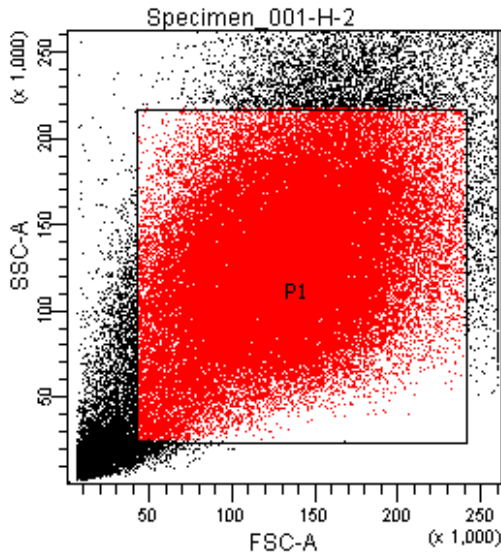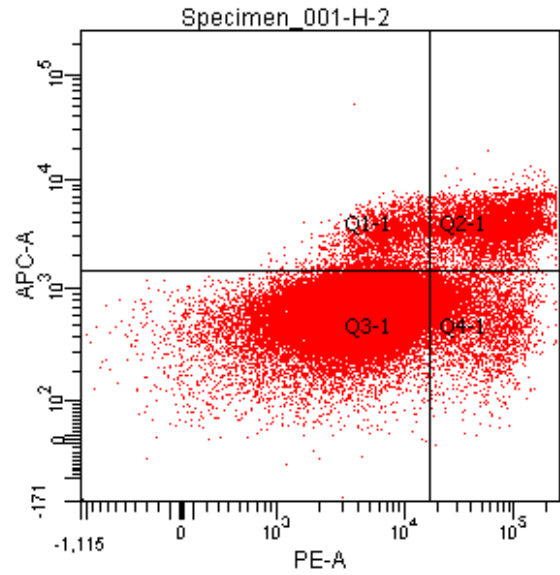

| Tube: H-2    |         |         |        |
|--------------|---------|---------|--------|
| Population   | #Events | %Parent | %Total |
| ■ All Events | 100,000 | ###     | 100.0  |
| ■ P1         | 75,211  | 75.2    | 75.2   |
| ☒ Q1-1       | 2,309   | 3.1     | 2.3    |
| ☒ Q2-1       | 5,365   | 7.1     | 5.4    |
| ☒ Q3-1       | 62,903  | 83.6    | 62.9   |
| ☒ Q4-1       | 4,634   | 6.2     | 4.6    |

Supplement: Supplemental Information 1 [file peerj-09-11401-s001.zip › Raw data or code/s-Fig2B. NAM&H2O2 Flow cytometry-based Annexin V7-AAD assay/H2O2.pdf]

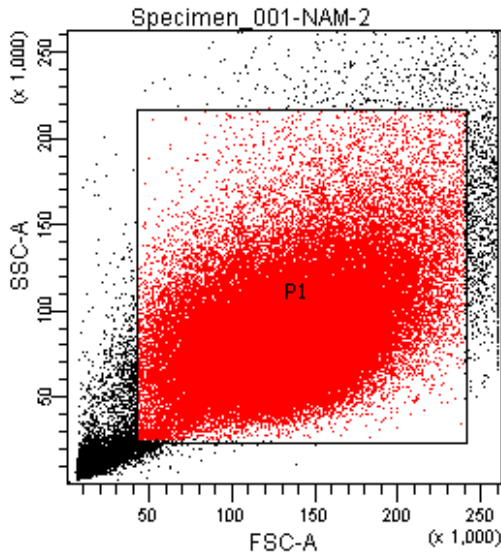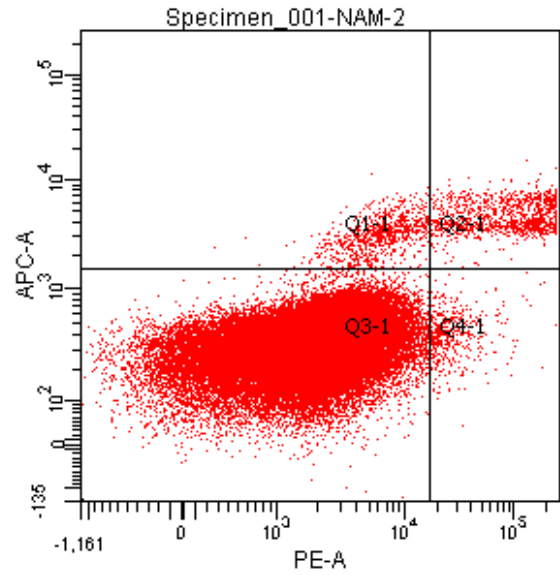

| Tube: NAM-2  |         |         |        |
|--------------|---------|---------|--------|
| Population   | #Events | %Parent | %Total |
| ■ All Events | 100,000 | ###     | 100.0  |
| ■ P1         | 85,050  | 85.0    | 85.0   |
| ☒ Q1-1       | 842     | 1.0     | 0.8    |
| ☒ Q2-1       | 1,194   | 1.4     | 1.2    |
| ☒ Q3-1       | 82,550  | 97.1    | 82.6   |
| ☒ Q4-1       | 464     | 0.5     | 0.5    |

Supplement: Supplemental Information 1 [file peerj-09-11401-s001.zip › Raw data or code/s-Fig2B. NAM&H2O2 Flow cytometry-based Annexin V7-AAD assay/NAM.pdf]

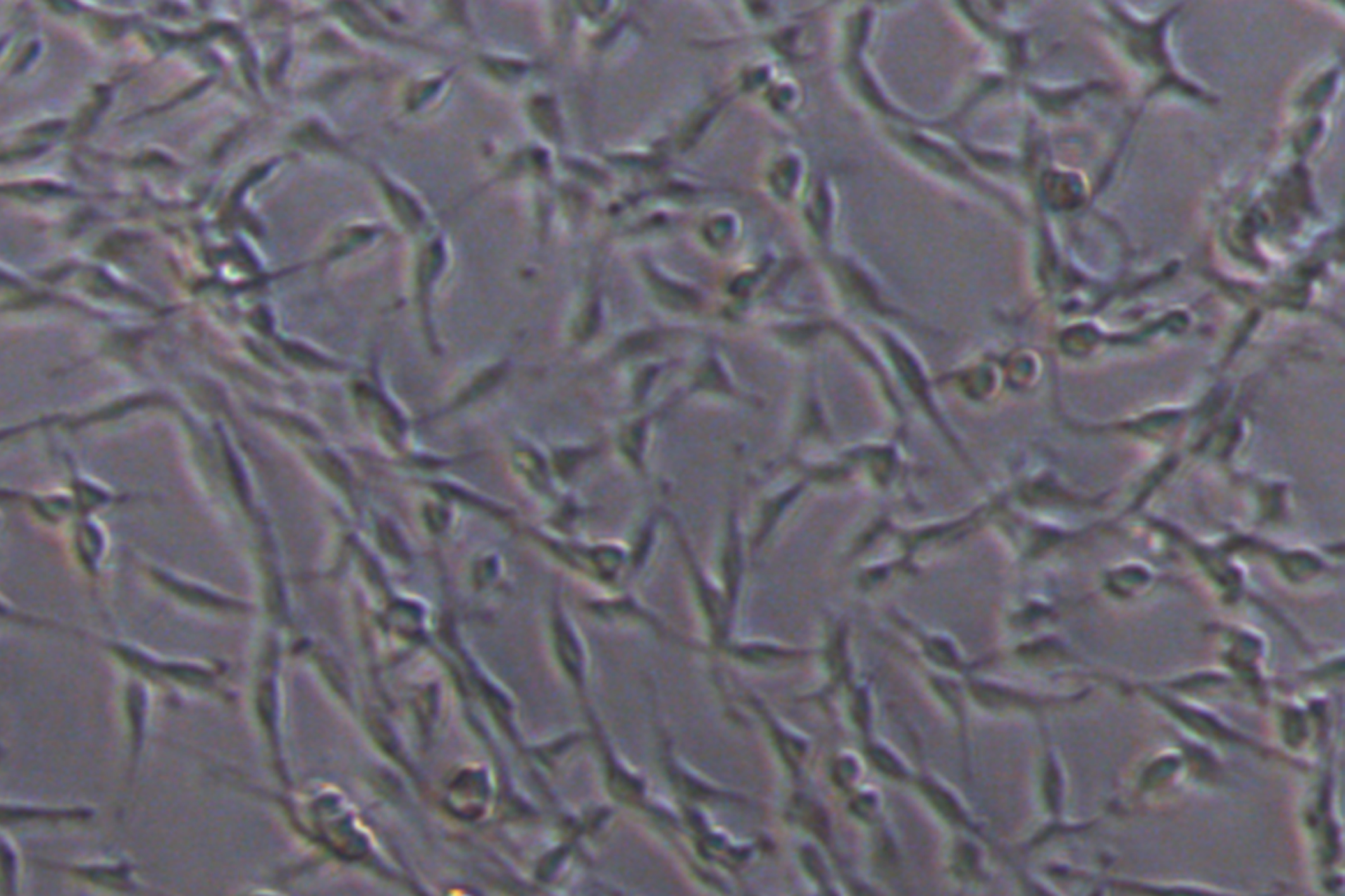

Supplement: Supplemental Information 2 [file peerj-09-11401-s002.png]

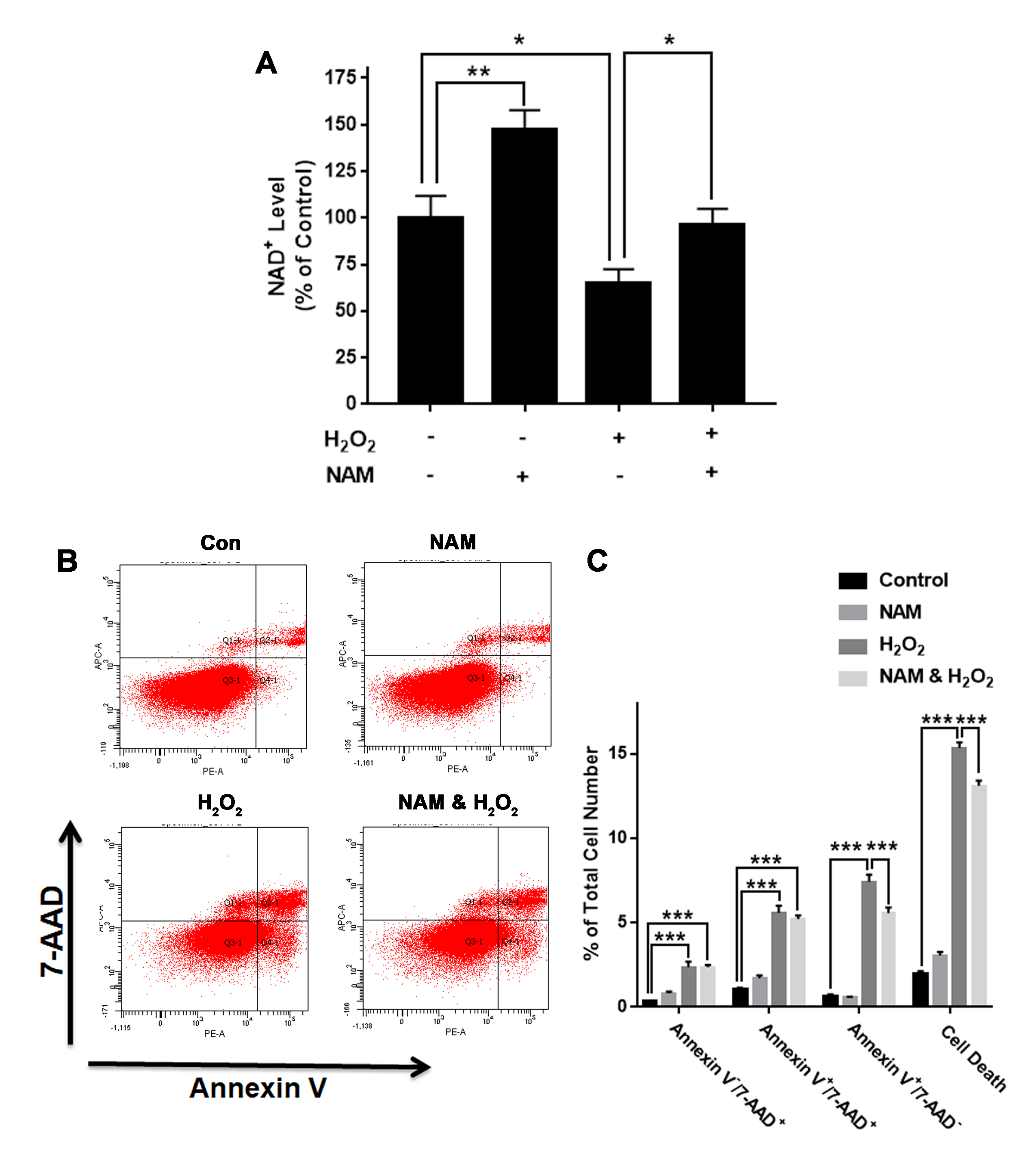

Supplement: Supplemental Information 3 — (A) NAM significantly attenuated the H2O2-produced decrease in the NAD+ levels of PC12 cells. (B, C) NAM significantly attenuated the H2O2-produced cell death. PC12 cells were pre-treated with 1 mM NAM for 1 h, and then co-treated with 0.3 mM H2O2 for 6 h (for NAD+ assay) or 23 h (for flow cytometry assay). The data were pooled from three independent experiments. N = 3. There were 3, 3 and 3 samples in these three independent experiments. *P < 0.05; **P < 0.01; ***P < 0.001. [file peerj-09-11401-s003.png]
